# Comprehensive and deep learning classification for analyses of the biological complexity of growth and biofilms of *Cobetia marina* under different temperature growths

**DOI:** 10.1371/journal.pone.0336575

**Published:** 2025-12-11

**Authors:** M. Alejandro Dinamarca, Claudia Ibacache-Quiroga, Karoll González-Pizarro, Jozef Kiseľák, Bastían Barraza-Morales, Benjamín León Schuffeneger, Milan Stehlík

**Affiliations:** 1 Centro de Micro-Bioinnovación, Universidad de Valparaíso, Valparaíso, Chile; 2 Escuela de Nutrición y Dietética, Facultad de Farmacia, Universidad de Valparaíso, Valparaíso, Chile; 3 Institute of Statistics, Universidad de Valparaíso, Valparaíso, Chile; 4 Institute of Mathematics, P.J.Šafárik University, Jesenná, Košice, Slovakia; Purdue University, UNITED STATES OF AMERICA

## Abstract

**Background:** Biological complexity represents a fundamental challenge in understanding microbial behavior, particularly when analyzing heterogeneous data from bacterial growth and biofilm formation. Traditional models often reduce data dispersion at the cost of losing biological interpretation, limiting their applicability to real-world scenarios.

**Methods:** We investigated biological complexity using *Cobetia marina* as a model organism, conducting comprehensive studies on growth kinetics and biofilm formation across a wide temperature range (8 ^∘^C to 41 ^∘^C). Mutant strains were generated using pUTmini-Tn5-Km transposons to study phenotypic variations independent of environmental variables. High-throughput screening was performed using 96-well microplates to ensure adequate experimental replication. Data analysis employed advanced mathematical techniques, including semi-automatic bi- and tri-classifiers, a novel fractional derivative method for growth classification, and SPOCU (Scaled Polynomial Constant Unit) for biofilm formation.

**Results:** We successfully developed classification systems to distinguish growth kinetics at minimum, optimal, and maximum temperatures. A neural network incorporating the SPOCU (Scaled Polynomial Constant Unit) transfer function demonstrated superior performance compared to conventional classifiers (SELU and RELU) in predicting biofilm production. The fractional derivative method proved effective in addressing key challenges in bi- and tri- classifier systems for temperature-dependent growth analysis.

**Conclusions:** This study demonstrates the effectiveness of advanced computational approaches in analyzing biological complexity. The integration of deep learning methods with comprehensive experimental design provides a robust framework for understanding microbial behavior under varying environmental conditions, with potential applications in biotechnology and environmental monitoring.

## 1 Introduction

Biological data and information often present both challenges and opportunities for the development of mathematics, statistics, and computer science. One of the primary challenges lies in interpreting the variability observed in biological models, experimental designs, and biological processes. To address this, various mathematical and statistical tools have been developed to describe and model biological behavior. However, most of these modeling strategies assume homogeneity and fail to account for biological variation. The observation and characterization of such variability have been crucial for the advancement of new mathematical and statistical approaches. For instance, several widely used statistical tests were originally developed by biologists: Fisher (experimental design and ANOVA), Pearson (correlation coefficient), Galton (regression to the mean and statistical correlation), Wright (path analysis and structural models), and Haldane (population genetics and statistical enumeration), see [1].

Today, the proliferation of biological data introduces new challenges for mathematics and statistics, particularly in understanding life phenomena from convergent disciplinary perspectives. In this context, one of the challenges is to assess the significance of data variation or dispersion resulting from the behavior of biological systems under different conditions and in response to specific variables. For example, understanding the stochasticity or randomness of data associated with acclimatization or adaptation phenomena under changing selection pressures can be highly relevant for studying processes across multiple scales. Advances in experimental methods, miniaturization, and real-time measurement technologies have enabled more precise and replicable acquisition of kinetic and process data. For instance, traditional microbial growth kinetics using microtiter plates with up to 96 wells, combined with spectrophotometric equipment with incubation capabilities, now highlight the significance of data dispersion and heterogeniety and open opportunities for developing new mathematical and statistical models.

In this scenario, studying biological phenomena such as bacterial growth kinetics and biofilm formation in response to changes in environmental selection pressures, like temperature, provides a suitable framework for developing advanced mathematical and statistical models and tools. Despite these advances in the analysis of bio-based data, there are still several mathematical and statistical challenges in analyzing biological data and their variability, and in understanding life phenomena through integrative approaches. For example, quantifying the stochasticity of acclimatization or adaptation processes under specific selection pressures remains a critical issue.

Biofilms are complex biological structures composed of microorganisms and biomolecules embedded in an extracellular matrix [2–4]. They can float or adhere to living or inert surfaces, causing substantial economic losses estimated at USD $5 trillion annually [4]. Conversely, biofilms play essential roles in aquatic and soil ecosystems and have been exploited for agricultural applications, pollutant bioindication, and biofiltration [5–8].

*Cobetia marina* is a ubiquitous marine bacterium capable of forming biofilms and inhabiting diverse marine environments, including the water column, macroalgae surfaces, and crustacean microbiota [9]. This microorganism is notable for its biofilm-forming ability and is widely used to evaluate new surfaces and biofilm-inhibiting compounds [10–14]. Its capacity to grow and form biofilms across a wide temperature range necessitates robust mathematical and statistical analyses to capture its biological complexity.

Building on these considerations, this study focuses on how variations in growth and biofilm formation by *Cobetia marina* under different conditions generate data with diverse levels of variability, offering valuable insights for mathematical, statistical, and computational modeling.

To address this, in Sect 2.2 we first develop a semi-automatic classifier for bacterial growth temperatures and outline the principles for constructing models capable of categorizing new and unknown growth curves into predefined temperature classes. Recognizing the limitations of standard numerical derivatives—which have proven inadequate for this purpose—we introduce a novel approach based on fractional derivatives to differentiate growth kinetics at minimum, optimal, and maximum temperatures. The application of fractional calculus to biological systems has emerged as a powerful mathematical tool for modeling complex biological phenomena. Fractional derivatives provide a more accurate representation of memory effects and hereditary properties in biological systems compared to classical integer-order derivatives. As discussed in Sect 2.4, we propose fractional derivatives as a robust alternative for distinguishing typical from atypical temperature responses, a critical challenge in bi-classifier and tri-classifier systems.

In Sect 2.5.1, we apply a deep learning analysis using SPOCU with memory to predict biofilm formation at different temperatures. Our results demonstrate that a neural network utilizing the SPOCU activation function [15] outperforms others in predicting biofilm production, both for the wild-type strain and mutant strains, highlighting the value of biological data variation under realistic conditions to develop robust statistical models and semi-automatic classification tools. Furthermore, we propose that the fractional order of growth serves as a biologically interpretable indicator of the system’s memory and the bacteria’s environmental response.

## 2 Material and methods

### 2.1 Data source, specification and collection

#### 2.1.1 Strain, culture conditions and biofilm formation.

This study utilized the *Cobetia marina* strain MM1IDA2H-1 (CECT 7764) as a model for biofilm formation [16]. It was cultured in a marine medium containing 0.1% yeast extract, 0.5% tryptone (casein peptone) and seawater. Biofilm formation and bacterial growth were evaluated at 8 ^∘^C, 16 ^∘^C, 20 ^∘^C, 25 ^∘^C, 30 ^∘^C, 35 ^∘^C, 38 ^∘^C, and 41 ^∘^C temperatures using optical density measurements at 600 nm for growth. Plates were incubated at each selected using incubators or a spectrophotometer equipped with incubation mode and temperature control (Tecan, model Infinite 200Pro serial number: 1310009538). For biofilm formation, the protocol uses 96-well microplates and a spectrophotometer with temperature control. Microplates were incubated in temperature-controlled environments with precise temperature regulation (±0.1 ^∘^C). Temperature gradients were established to cover the complete physiological range of *Cobetia marina*, from minimum growth temperature (8 ^∘^C) through optimal conditions (35 ^∘^C) to maximum tolerance (41 ^∘^C). After incubation, plates were stained with crystal violet, rinsed, and treated with acetic acid in ethanol to measure absorbance [17]. After growth periods, planktonic cells were removed by gentle washing with phosphate-buffered saline (PBS). Wells were then stained with 0.1% crystal violet solution for 15 minutes at room temperature. The study analyzed 285 culture replicates per temperature, with a total of 4560 data points collected. Data is available as supplementary material. Data output analyzed are growth at different times with optical densities at 600 nm, and biofilms with optical densities at 540 nm.

#### 2.1.2 Obtaining a mutant library of *Cobetia marina.*

To generate different growth kinetics phenotypes, a mutant library of *Cobetia marina* was created through random chromosomal insertion mutagenesis using transposable elements. Briefly, a mini transposon with kanamycin resistance was used, involving triparental conjugations between *Escherichia coli* carrying the plasmid pUT-mini-Tn5-Km, *Escherichia coli* containing a helper plasmid for conjugation functions, and the bacterium *Cobetia marina* as the plasmid recipient. A total of 90–96 mutants were obtained and characterized for their growth kinetics. The mutants were grown to generate growth kinetics data at 35 ^∘^C and biofilm formation at the same temperature.

### 2.2 On the construction of a classifier and bi-classifier for growth kinetics of *Cobetia marina* growing at different temperatures

Here, we are utilizing the following biologically motivated auxiliary variables:

1. The standard derivative (slope) of the growth curve.
2. The total sum of all optical densities (as an integral).
3. For each assay, individual values of OD as a time series (for comparison with the model curve OD values in the class categories Minimum, Optimal, Maximum).

To identify and classify new curves, a selection of parameters has been made to classify the data of the different growth temperatures into three categories:

- **Minimal:** 8 ^∘^C to 20 ^∘^C, using the bacterial growth at 8 ^∘^C as a model.
- **Optimal:** 24 ^∘^C to 37 ^∘^C, using the bacterial growth at 33 ^∘^C as a model.
- **Maximal:** 38 ^∘^C to 41 ^∘^C, using the bacterial growth at 41 ^∘^C as a model.

The model growth temperatures were used to define the pattern for their corresponding category. For each temperature, data from different growth trials of *Cobetia marina* were collected over 48 hours.

### 2.3 Procedure

The following methodologies were implemented:

#### 2.3.1 Bi-classifier.

Each test, corresponding to a specific temperature condition, was initially evaluated by calculating its correlation with each of the previously defined model temperature curves. For each test, the model curve that showed the highest degree of correlation was selected. This maximum correlation value was used as the first criterion for classifying the test within the defined temperature categories. This process constitutes the first part of the bi-classifier’s operational structure. Subsequently, a second evaluation criterion was applied to each test: the measurement of proximity to each of the model curves. This proximity was determined by calculating the mean squared error (MSE) between the test curve and each model curve. For each test, the model curve with the lowest MSE value was selected. Therefore, the mean squared error was considered the second key parameter shaping the bi-classifier’s structure. Once the correlation coefficients and mean squared errors were obtained for all tests, a detailed analysis of the observed trends was carried out. Based on this analysis, specific rules were established to govern the final decision-making process of the bi-classifier. These rules are presented in Table 2 in Sect 3.1.

#### 2.3.2 Tri-classifier.

The existing binary classifier has been extended to a ternary classification approach capable of distinguishing between minimum, optimal, and maximum temperature categories. In order to achieve a more accurate classification for the 40 ^∘^C temperature condition, which, as observed in Table 5, exhibited the highest number of false positives, reaching 100%- an additional rule was incorporated into the classification system.

- If the total sum of the curve falls within the range [95 – 114.7], then the test is not classified as belonging to 40 ^∘^C.
- If the variance of the curve falls within the range [0.022 – 0.052] and the sum of the curve’s values falls within the range [87.8 – 114.9], then the test is classified as corresponding to 40 ^∘^C.
- In any other case, the test is not classified as 40 ^∘^C.

This new rule was specifically applied to tests where the initial classification by the bi-classifier resulted in “Optimal – Optimal," and whose data corresponded to nearby temperatures, namely: 24 ^∘^C, 26 ^∘^C, 30 ^∘^C, 34 ^∘^C, 35 ^∘^C, 36 ^∘^C, 37 ^∘^C, 38 ^∘^C, and 40 ^∘^C.

However, despite this refinement, false positives were still observed, reaching 4.7% of the total cases. This is because there are subsets of data from other temperatures that, in some cases, fall within the predefined ranges for 40 ^∘^C, thereby generating an overlap of characteristics that makes it difficult to achieve completely accurate separation. The Table 3 presents the number of false positives detected during this validation process of the bi-classifier. Finally, both classifiers can be highly useful for identifying the temperature to which the bacteria are exposed, by analyzing the behavior of their growth curves and directly comparing them with previously trained model curves. This approach enables successful classification with a low percentage of false positives. The results of both the bi-classifier and tri-classifier can be found in Sect 3.1

### 2.4 Fractional-derivative-based classification

Fractional derivatives are often superior to standard derivatives for growth classification because they better capture the complexities and non-integer order characteristics of growth processes. They allow for a more nuanced understanding of how growth is influenced by past events and internal dynamics, which standard derivatives might miss. They can incorporate memory and history, leading to improved accuracy. In our experiments, standard derivatives failed to classify the data accurately, and we have not been able to find good results by adapting several empirical classification experiments, such as including the number of negative standard derivatives in classification rules. These issues primarily motivated fractional classifications, where local property is modeled by global fractional derivative property.

Fractional derivatives are non-local, so, for example, the half derivative (1/2-th derivative) cannot have a local meaning like tangent or curvature. It would have to take into account the properties of the curve over a large extent. We focus on the Caputo-Fabrizio (CF) fractional derivative, which is a type of non-singular fractional derivative. This derivative avoids the singular kernel found in the classical Caputo derivative by using an exponential kernel instead of a power-law kernel (like Riemann–Liouville or Caputo), i.e., the singular kernel $K(t,s)=(t\;-\;s){\;}^{-\kappa }$ is replaced by the kernel $K(t,s)=e^{-\frac{\kappa }{1-\kappa }(t-s)}$. Notice also that one replaces the constant $\frac{1}{\Gamma (1-\kappa )}$ by $\frac{1}{1-\kappa }$ ( or even by $\frac{M(\kappa )}{1-\kappa }$ with a normalization function M:M(0)=M(1)=1).

For a real sufficiently smooth function *f*, the κ-th CF fractional derivative, κ∈[0,1), is given by


$${𝒟}^{\kappa }f(t)=\frac{1}{1-\kappa }\int_{0}^{t}e^{-\frac{\kappa }{1-\kappa }(t-s)}f^{\prime }(s)ds,$$

see [18]. As κ→1, the exponential kernel tends to the Dirac delta function δ(t−τ), and thus, the CF derivative converges (not pointwise but in the distributional (or weak) sense) to the classical first-order derivative:


$$\lim_{\kappa \to 1^{-}}D^{\kappa }f(t)\overset{w}{=}f^{\prime }(t)$$

As $\kappa \to 0^{+}$, the exponential term becomes exp(0)=1, and the kernel becomes constant. Then, the CF derivative reduces to:


$$\lim_{\kappa \to 0}D^{\kappa }f(t)=\int_{0}^{t}f^{\prime }(\tau )\;d\tau =f(t)-f(0)$$

**Fig 1:**
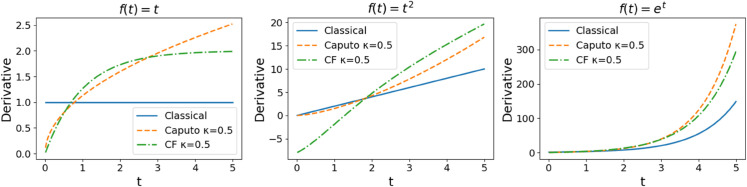
Graphs of classical, Caputo, and CF derivatives of basic functions.

The CF derivative avoids singularities at *t* = 0, leading to improved numerical stability. Table 1 and Fig 1 highlight key differences in how classical, Caputo, and CF derivatives behave on elementary functions. For power functions such as f(t)=t or *t*^2^, the Caputo derivative introduces a fractional power-law scaling, which reflects long-range memory effects: earlier values of the function heavily influence the current rate of change. In contrast, the CF derivative uses an exponential kernel, which results in a smoother memory decay and avoids singularities at *t* = 0, making it more numerically stable. CF avoids infinite memory at the start, which is more realistic for many natural systems. It models exponentially fading memory, meaning that recent states of the system matter more than older ones—a short-term memory effect.

**Table 1:** Comparison of classical, Caputo, and CF derivatives for basic functions.

Function f(t)	Classical	Caputo	CF
*t*	1	$\frac{t^{1-\kappa }}{\Gamma (2-\kappa )}$	$\frac{1-e^{-\frac{\kappa }{1-\kappa }t}}{1-\kappa }$
*t* ^2^	2*t*	$\frac{2t^{2-\kappa }}{\Gamma (3-\kappa )}$	$\frac{2t}{1-\kappa }-\frac{2(1+\frac{\kappa }{1-\kappa }t)e^{-\frac{\kappa }{1-\kappa }t}}{(1-\kappa ){\;}^{2}}$
$e^{\lambda t}$	$\lambda e^{\lambda t}$	$\lambda^{\kappa }e^{\lambda t}$	$\frac{\lambda (1-e^{-\frac{\kappa }{1-\kappa }t})}{1-\kappa }e^{\lambda t}$

The corresponding fractional integral of a sufficiently smooth function *g* is

$${ℐ}^{\kappa }g(t)=(1-\kappa )[g(t)-g(0)]+\kappa \int_{0}^{t}g(s)\;ds$$ (1)

and we have that

$${ℐ}^{\kappa }{𝒟}^{\kappa }f(t)=f(t)+c,$$ (2)

where *c* is an arbitrary constant. If n≥1, and κ∈[0,1] the (κ+n)–th fractional derivative operator ${𝒟}^{\left(\kappa +n\right)}$ is defined as the composition( or multiplication) ${𝒟}^{\left(\kappa +n\right)}:={𝒟}^{\kappa }{𝒟}^{n}={𝒟}^{\kappa }\frac{d^{n}}{dt^{n}}$. Since we have that


$${𝒟}^{\kappa }f(t)=\frac{1}{1-\kappa }\int_{0}^{t}e^{-\frac{\kappa }{1-\kappa }(t-s)}f^{\prime }(s)ds=$$

$$=\frac{1}{1-\kappa }\left(f(t)-e^{-\frac{\kappa }{1-\kappa }t}f(0)\right)-\frac{\kappa }{(1-\kappa {)}^{2}}\int_{0}^{t}e^{-\frac{\kappa }{1-\kappa }(t-s)}f(s)ds$$

we can use a numerical approach that gives us a value at time *t*_*i*_ for κ∈(0,1)


$$\frac{1}{1-\kappa }\left(f(t_{i})-f(t_{0})e^{-\frac{\kappa }{1-\kappa }t_{i}}\right)-\frac{\kappa \;e^{-\frac{\kappa }{1-\kappa }t_{i}}}{(1-\kappa {)}^{2}}\sum_{j=0}^{i}f(t_{j})\;e^{\frac{\kappa }{1-\kappa }t_{j}}(t_{j+1}-t_{j}).$$

But we need a fractional parameter to be in the interval [1,2]–we call it fractional order and denote it as *η*. Thus we used numerical analogue to ${𝒟}^{\kappa +1}f(t)$ by ${𝒟}^{\eta }f(t):=\frac{1}{1-\kappa }\left(f^{\prime }(t)-f^{\prime }(0)e^{-\frac{\kappa }{1-\kappa }t}\right)\;-\;\frac{\kappa }{1-\kappa }{𝒟}^{\kappa }f(t),$ i.e. *η* can be understood (in some sense) as κ+1. We have computed such numerical derivatives on real data in a specific order. The results can be found in Sect 3.3.

#### 2.4.1 Standard differential modeling.

Here we mention well-known models. We begin by considering a modification of the *generalized logistic differential equation* (also known as *Richard’s equation*):


$$y^{\prime }(t)=(y(t)-A)\;F(y(t);A,\alpha ,\nu ),$$

where the proliferation rate *F* is defined as


$$F(z;A,\alpha ,\nu )=\alpha \left(1-{\left(\frac{z-A}{K-A}\right)}^{\nu }\right).$$

Logistic-type growth models are widely used in biological, ecological, and medical contexts due to their flexibility and interpretability. Among them, the generalized logistic model–also known as Richard’s equation–offers a tunable growth profile that interpolates between classical logistic and Gompertz dynamics. Here

- *A* is the lower asymptote (often *A* = 0),
- K≥0 is the carrying capacity (upper asymptote, i.e. y(t)≤K),
- ν≥0, α≥0 are parameters to be estimated.

If we set $\tilde{G}(z)=\alpha (z-A)\left(1-\left(\frac{z-A}{K-A}\right){\;}^{\nu }\right)$, then its basic properties are

- $\tilde{G}(A)=\tilde{G}(K)=0$;
- achieves maximum at $z^{*}=A+(K-A)\left(\frac{1}{\nu +1}\right){\;}^{\frac{1}{\nu }}$ on [A,K];
- increasing first, then decreasing as y→K, similar to logistic or beta-growth models;
- generalizes unimodal growth function, shaped like a skewed parabola depending on ν;

The parameter ν controls the location of maximum growth, and αν represents the overall growth rate. This model includes the classical logistic model when ν=1, and converges to a Gompertz-type curve as $\nu \to 0^{+}$ with α=O(1/ν). The solution is given by

$$y(t)=A+\frac{K-A}{{\left(1+Qe^{-\alpha \nu (t-t_{0})}\right)}^{1/\nu }},\;Q={\left(\frac{K-A}{y(t_{0})-A}\right)}^{\nu }-1.$$

- For ν=1: we recover a modified logistic model.
- For $\nu \to 0^{+}$ and α=O(1/ν): we recover a modified Gompertz model:
  $$y^{\prime }(t)=\tilde{\alpha }\ln\left(\frac{y(t)-A}{K-A}\right)(y(t)-A).$$ (3)

While the classical and generalized logistic models offer excellent fits for many systems, they do not account for memory effects or anomalous growth dynamics. In what follows, we introduce a fractional generalization that incorporates such effects.

### 2.5 Neural network using SPOCU classifier

The results demonstrated a consistent decrease in RMSE with the use of SPOCU, confirming its superiority over other activation functions as network complexity grew. The experiments were implemented in Jupyter Notebooks using Python 3.0, and executed on a machine with an AMD Ryzen 5 3550H processor and 8GB of RAM. The SPOCU function was implemented using an open-source version available on GitHub.

A total of 20 experiments were conducted, comparing the performance of models using SPOCU and ReLU activation functions. Key metrics such as Loss, Validation Loss, total processing time, and processing time per epoch were recorded for each configuration. The results are in Sect 3.5.

#### 2.5.1 Classification of biofilm formation under different growth temperatures.

Here we present the modeling approach for classifying biofilm according to different temperatures in the bacterium *Cobetia marina*. We focus on identifying patterns in biofilm production using neural networks, utilizing a biologically motivated ratio as the dependent variable. The aim is to distinguish between two categories: high and low biofilm production, or three categories: minimal, optimal, and maximal temperature.

To model biofilm production, we define a random variable as the ratio of two positive variables: ABS540 and ABS600. This biologically motivated ratio is expressed as:

$$R=\frac{\text{ABS540}}{\text{ABS600}}$$ (4)

Here, *R* represents the ratio between the absorbance at 540 nm (indicative of the amount of biofilm) and the absorbance at 600 nm (indicative of biomass). This ratio provides a biologically motivated measure of biofilm production relative to total biomass and is the dependent variable in our model.

To obtain the *classification of the ratio*, the median of the *R* values is used to establish two categories: *Maximal* (high biofilm production) and *Minimal* (low biofilm production). Values above the median are classified as *Maximal*, while values below the median are classified as *Minimal*. For the three-category classification, the data is divided using quartiles: values below the first quartile are categorized as Minimal, values between the first and third quartiles as Optimal, and values above the third quartile as Maximal. Furthermore, to avoid potential distortions due to differences in variable scales, the ratio *R* is standardized before being used in the neural networks.

Three **neural network models** are proposed to find the best architecture for classifying high and low biofilm production. The three networks share the following characteristics:

- Two hidden layers. The first has 32 neurons and the second has 16 neurons.
- An output layer using the *sigmoid activation function for binary classification or softmax for three-class classification.*
- The use of *Stochastic Gradient Descent (SGD)* optimizer with a learning rate of 0.0001.

The main difference between the models lies in the activation function used in the second hidden layer:

- Neural Network 1: ReLU activation function.
- Neural Network 2: SELU activation function.
- Neural Network 3: SPOCU activation function, with parameters α=2, β=0.75, γ=2, and *c* = 2.

The results can be found in Sect 3.5.1.

#### 2.5.2 Classification of biofilm formation in response to a biofilm inhibitor.

To investigate the effects of inhibitor concentrations on the growth and biofilm formation of *Cobetia marina*, we employed deep learning classification techniques.

As a first step in data preprocessing, the ratio defined in Equation 4 was normalized using the RobustScaler method.

To enable binary classification of biofilm production in mutant strains, the biofilm production ratio *R* was categorized according to inhibitor concentration, dividing the samples into two classes: Minimal (less than 0.00001) and Maximal (equal to or greater than 0.00001).

For model development, three neural network architectures were implemented, each differing in the activation function applied in the second hidden layer:

1. ReLU
2. SELU
3. SPOCU

All models shared a common structure consisting of a single input neuron representing the normalized biofilm production, a first hidden layer with 32 ReLU neurons, a second hidden layer with 16 neurons (using ReLU, SELU, or SPOCU), and an output layer. The output layer comprised either a single sigmoid neuron for binary classification or three softmax neurons for multi-class classification.

The dataset was randomly split using a fixed seed to ensure reproducibility. No stratification or domain-specific criteria were applied. This approach preserved an unbiased distribution of growth and biofilm replicates while reflecting the natural heterogeneity of the data. During training, all models were optimized using the Adam algorithm with a learning rate of 0.001. The loss function employed was either binary or categorical cross-entropy, depending on the classification task. Training was carried out over 100 epochs with a batch size of 32, reserving 20% of the data for validation. This configuration represents a commonly used baseline for self-normalizing networks and was chosen to balance model capacity with computational feasibility. In our case, it provided sufficient flexibility to capture the complexity of growth kinetics and biofilm variability without overfitting, while remaining efficient for the dataset size (4560 data points).

No hyperparameter tuning was based on validation results. Instead, the validation data provided an unbiased check of generalization, while the independent test set was reserved for final evaluation. This strategy emphasized robustness and biological interpretability, with parameters refined through preliminary experiments rather than exhaustive hyperparameter optimization.

To evaluate model generalization, a separate test set comprising 20% of the data was used. Performance metrics included accuracy, precision, recall, and F1 score. Additionally, we analyzed training and validation loss and accuracy curves, along with confusion matrices, to provide a comprehensive overview of model performance. Detailed results are presented in Sect 3.5.2.

#### 2.5.3 *Cobetia marina* random mutants derived by transposable element mutagenesis.

This section investigates the classification of biofilm formation in *Cobetia marina* mutant strains using deep learning. Approximately 400 mutant clones of *Cobetia marina* were obtained through random mutagenesis using miniTn5 transposable elements. The growth kinetics of each mutant were evaluated. Following data normalization with RobustScaler, neural network models were trained to categorize biofilm production based on the ratio (R) of mutant strains. Two distinct classification strategies were employed: The first strategy utilized a median-based approach, dividing the data into two classes: *Minimal* (ratios below the median) and *Maximal* (ratios equal to or above the median). The second strategy implemented a quartile-based approach, segmenting the data into three categories: values $\leq Q_{1}$ were labeled as *Minimal*, values between *Q*_1_ and *Q*_3_ as *Optimal*, and values >*Q*_3_ as *Maximal*.

Similar to Sect 2.5.2, the neural network architecture consisted of an input layer, two hidden layers (32 ReLU neurons in the first, and 16 neurons with ReLU, SELU, or SPOCU in the second), and an output layer (sigmoid for binary, softmax for multi-class). Model training utilized the Adam optimizer (learning rate 0.001), binary or categorical cross-entropy loss, a batch size of 32, and 100 epochs, with 20% of the data reserved for validation. Model performance was evaluated on a separate 20% test set using accuracy, precision, recall, and F1 score, along with analysis of loss/accuracy curves and confusion matrices (results detailed in 3.5.2).

## 3 Results

### 3.1 Classifiers procedures for growth kinetics according to temperatures

#### 3.1.1 Bi-classifier analysis.

During the evaluation of the results, specific classification rules were established for the bi-classifier. These rules, which guided the classification based on the correlation and RMSE metrics, are summarized in Table 2.

**Table 2:** Decision rules for the bi-classifier.

Correlation	RMSE	bi-classifier Output
Minimal	Minimal	Minimal
Minimal	Maximal	Minimal
Optimal	Optimal	Optimal
Optimal	Maximal	Optimal
Minimal	Optimal	Maximal

Combinations not shown in the table above were not encountered or reported in the results. Despite the overall strong performance of the bi-classifier, several false positives were observed. The temperature-wise distribution of these false positives is presented in Table 3. Additional details can be found in the Excel file “Original bi-classifier Data Table".

**Table 3:** False positives by temperature (bi-classifier).

Temperature (^∘^C)	False Positives	Total Cases
10	0	25
12	0	25
16	0	25
18	2	25
20	5	25
24	0	25
26	0	25
30	0	25
34	0	25
35	0	25
36	0	25
37	0	10
38	2	10
39	0	10
40	10	10

The average classification results per temperature, based on correlation and RMSE metrics, are summarized in Table 4. The corresponding bi-classifier outputs were inferred using the decision rules.

**Table 4:** Average classification outcomes by temperature (bi-classifier).

Temperature (^∘^C)	Correlation	RMSE	bi-classifier Output
10	Minimal	Minimal	Minimal
12	Minimal	Minimal	Minimal
16	Minimal	Maximal	Minimal
18	Minimal	Maximal	Minimal
20	Minimal	Maximal	Minimal
24	Optimal	Optimal	Optimal
26	Optimal	Optimal	Optimal
30	Optimal	Optimal	Optimal
34	Optimal	Optimal	Optimal
35	Optimal	Optimal	Optimal
36	Optimal	Optimal	Optimal
37	Optimal	Optimal	Optimal
38	Minimal	Optimal	Maximal
39	Minimal	Optimal	Maximal
40	Optimal	Optimal	Optimal

#### 3.1.2 Tri-classifier analysis.

Table 5 reports the number of false positives identified by the tri-classifier across different temperatures. The complete dataset and additional metrics are available in the Excel file “Original Tri-Classified Data Table".

**Table 5:** False positives by temperature (tri-classifier).

Temperature (^∘^C)	False Positives	Total Cases
10	0	25
12	0	25
16	0	25
18	0	25
20	0	25
24	4	25
26	5	25
30	0	25
34	0	25
35	0	25
36	0	25
37	0	10
38	0	10
39	0	10
40	6	10

### 3.2 Theoretical results for construction of fractional classifier

#### 3.2.1 Proposed model with fractional derivative.

In this work, we propose incorporating fractional-order memory into the model. Specifically, we retain the classical inner derivative but replace the outer derivative in a second-order formulation with the CF fractional derivative ${𝒟}^{\eta }$. First, we differentiate Richard’s equation using a non-fractional derivative. Then we replace the operator $\frac{d^{2}}{dt^{2}}$ with the operator ${𝒟}^{\eta }$, i.e., we change only the “outer" derivative of the composition $\frac{d}{dt}\;\circ \;\frac{d}{dt}$ to obtain ${𝒟}^{\kappa }\circ \frac{d}{dt}$. This leads to a model capturing fractional acceleration-like behavior.

$${𝒟}^{\eta }y(t)=\frac{d}{dt}\left\{(y(t)-A)\;\alpha \left(1-{\left(\frac{y(t)-A}{K-A}\right)}^{\nu }\right)\right\}.$$ (5)

This fractional model blends nonlinear logistic-type growth with short-term memory effects, making it suitable for phenomena such as:

- Biological growth with latent or delayed phases,
- Immune responses,
- Short-lived environmental or resource constraints.

Here, η∈[1,2]:η>1 reflects a second-order-like behavior with memory, modeling not only the growth rate but also how that rate has evolved—akin to a *memory-weighted acceleration*. We can say that it is a system with second-order memory, like “fractional inertia". The system effectively “remembers" both its velocity, typically considered to be $\mu_{\text{MAX}}$, and how that velocity has changed. Velocity is often biologically understood as the growth rate, $\mu_{\text{MAX}}$, or maximal doubling time in experiments. It can better represent systems where the past has a diminishing influence and can describe delayed or damped responses more realistically than classical models since the growth depends on both the current rate and the memory of how that rate has evolved. Growth is influenced by past behavior, but this influence decays exponentially. This is crucial in modeling biological growth with latent phases.

#### 3.2.2 Integral reformulation.

In what follows, we set *t*_0_ = 0. To handle the fractional dynamics, we follow a strategy inspired by [19], where only a specific case was solved - the fractional logistic ordinary differential equation. We solved a more general fractional DE by transforming it into a classical ODE. Consider

$${𝒟}^{\kappa }y(t)=G(t,y(t)),$$ (6)

then, if *y* is a solution of (6), integrating we get ${ℐ}^{\kappa }{𝒟}^{\kappa }y(t)={ℐ}^{\kappa }Y(t)$, where Y(t)=G(t,y(t)). Thus using (1) and (2) we obtain


$$y(t)-y(0)=(1-\kappa )[Y(t)-Y(0)]+\kappa \int_{0}^{t}Y(s)\;ds$$

and after differentiation, we have

$$y^{\prime }(t)=(1-\kappa )Y^{\prime }(t)+\kappa Y(t)$$ (7)

and therefore


$$y^{\prime }(t)=(1-\kappa )\left[\frac{\partial G}{\partial t}(t,y(t))+y^{\prime }(t)\frac{\partial G}{\partial y}(t,y(t))\right]+\kappa \;G(t,y(t)).$$

for a given *G*, this is a first-order ordinary differential equation (ODE). Now, if *G* does not explicitly depend on *t* (which is the standard assumption of autonomous dynamics), then we have a separable ODE.

**Remark 3.1.** Thus, in the case of the κ-th fractional generalized logistic differential equation (Richard’s equation)


$${𝒟}^{\kappa }y(t)=(y(t)-A)\;\alpha \left(1-{\left(\frac{y(t)-A}{K-A}\right)}^{\nu }\right),$$

where *G* is involved also in the right-hand side in (5). Consequently, we obtain a separable equation implying

$$-\frac{2}{\nu }\;arctanh[1-2((A-y)/(A-K){)}^{\nu }]+(\kappa -1)\ln|F(y;A,\alpha ,\nu )|=\kappa t+C.$$ (8)

This equation provides the solution in analytic (implicit) form.

#### 3.2.3 Integral reformulation for fractional order.

If we assume the model *y*" = *G*(*t*, *y*), it could be generalized as ${𝒟}^{\eta }y=G(t,y)$. However, notice that due to the lack of commutativity, ${𝒟}^{\left(\kappa +n\right)}\neq {𝒟}^{\left(n+\kappa \right)}$, we should be careful, as it may not be the same as ${𝒟}^{\left(1+\kappa \right)}y=G(t,y)$, and the solutions of two such models would be totally different. In other words, the order *η* is not equivalent to the order 1+κ. However, our approach has better properties. Since we have $Y^{\prime }(t)$ on the right-hand side, we should emphasize that for the operator ${𝒟}^{\eta }$, we obtain an equation similar to equation (7).

From the definition of the derivative of order *η*, we have


$$y^{\prime }(t)-y^{\prime }(0)=(2-\eta )[Y^{\prime }(t)-Y^{\prime }(0)]+(\eta -1)\int_{0}^{t}Y^{\prime }(s)\;ds$$

and therefore equation


$$y^{\prime }(t)-y^{\prime }(0)=(2-\eta )[Y^{\prime }(t)-Y^{\prime }(0)]+(\eta -1)[Y(t)-Y(0)]$$

or

$$y^{\prime }(t)=(2-\eta )Y^{\prime }(t)+(\eta -1)Y(t)+C$$ (9)

which differs from (7) only by a constant. Now, suppose we have the model ${𝒟}^{\left(1+\kappa \right)}y=\frac{d}{dt}G(t,y(t))$ (e.g., our main model (5) but with ${𝒟}^{\left(1+\kappa \right)}y$ on the left-hand side). This directly implies the model ${𝒟}^{\kappa }y=G(t,y(t))+c$, i.e., ${𝒟}^{\kappa }y(t)=Y(t)+c$. This implies $y^{\prime }(t)=(1-\kappa )Y^{\prime }(t)+\kappa Y(t)+\kappa \;c$, which is equivalent to (9). Thus, we have proved the following theorem.

**Theorem 3.2.** *The CF derivative models* ${𝒟}^{\left(1+\kappa \right)}y=\frac{d}{dt}G(t,y(t))$ *and* ${𝒟}^{\eta }y=\frac{d}{dt}G(t,y(t))$ *are equivalent.*

In the following examples, we will also highlight the significant difference between linear and non-linear right-hand sides.

**Example 3.3** (Linear). Suppose the basal linear ODE is $y^{\prime }=y$, i.e., the case κ=1. If κ∈(0,1) then

- case κ implies ${𝒟}^{\kappa }y=y$, i.e., $y^{\prime }=(1-\kappa )y^{\prime }+\kappa y$, i.e., $y^{\prime }=y$ (we obtain basal ODE );
- case *η* implies ${𝒟}^{\eta }y=y^{\prime }$, i.e., $y^{\prime }=(1-\kappa )y^{\prime }+\kappa y+\tilde{c}$, i.e., $y^{\prime }=y+c$, i.e. $y^{\prime }=y+c$.

The difference between the first two equations yields the difference in their solutions $y=c_{2}\;e^{t}$ and $y=c_{1}+c_{2}\;e^{t}$. Here, the difference is only a constant *c*_1_.

**Example 3.4** (Non-linear). Suppose the basal non-linear ODE is $y^{\prime }=y^{2}$. If κ∈(0,1) then

- case κ implies ${𝒟}^{\kappa }y=y^{2}$, i.e., $y^{\prime }=2(1-\kappa )yy^{\prime }+\kappa y^{2}$;
- case *η* implies ${𝒟}^{\eta }y=y^{\prime }$, i.e., $y^{\prime }=2(2-\eta )yy^{\prime }+(\eta -1)y^{2}+\tilde{c}$.

However, if η→2 (κ→1), the first two equations are $y^{\prime }=y^{2}$ and $y^{\prime }=y^{2}\;+\;\tilde{c}$ (i.e., *y*" = 2*yy*′), but the difference in their solutions is more complex: $s=\frac{1}{c_{1}-t}$ and $s=\tan\left(\frac{c_{2}+t}{c_{1}}\right)/c_{1}.$

Notice that from (9), we obtain an integral equation, which is more stable for estimation and fitting.


$$y(t)-y_{0}=(2-\eta )[Y(t)-Y(0)]+(\eta -1)\int_{0}^{t}Y(s)\;ds+c\;t$$

and thus we have a model

$$y(t)-y_{0}=(2-\eta )\alpha [(y(t)-A)\left(1-{\left(\frac{y(t)-A}{K-A}\right)}^{\nu }\right)\;\;\;-(y_{0}-A)\left(1-{\left(\frac{y_{0}-A}{K-A}\right)}^{\nu }\right)]\;\;+(\eta -1)\alpha \int_{0}^{t}(y(s)-A)\left(1-{\left(\frac{y(s)-A}{K-A}\right)}^{\nu }\right)\;ds+c\;t$$ (10)

#### 3.2.4 Parameter estimation procedure.

To model bacterial growth, each experimental time series of optical density readings was fitted using the nonlinear dynamic model governed by the ODE (9) with a fractional-like nonlinear growth term. The initial condition *y*_0_ and *K* (maximal value) were set from the experimental data, and the shape parameter ν, which controls the steepness and asymmetry of the saturation behavior, was fixed.

- Parameters κ, *α*, *A*, *c* are estimated via nonlinear least squares using scipy.optimize.curve_fit with the dogbox algorithm.
- The integral $\int_{0}^{t}\tilde{G}(y(s))\;ds$ is evaluated numerically using scipy.integrate.cumulative_trapezoid.

This integral formulation is numerically more stable than solving a differential model.

**Remark 3.5** (Estimation of *η* for fixed other parameters). Let $t_{1},\ldots ,t_{n}$ be observation times. For each *t*_*i*_, define


$$Z_{i}:=\Delta_{\tilde{G}}(t_{i})-I(t_{i}),\;Y_{i}:=y(t_{i})-y_{0}-I(t_{i})-ct_{i},$$

and set $\Delta_{\tilde{G}}(t)=\tilde{G}(y(t))-\tilde{G}(y_{0}),\;I(t)=\int_{0}^{t}\tilde{G}(y(s))\;ds$. Now, using the model prediction


$$\hat{y}(t_{i};\eta )=y_{0}+(2-\eta )\Delta_{\tilde{G}}(t_{i})+(\eta -1)I(t_{i})+ct_{i}.$$

The residual is $r_{i}(\eta )=y(t_{i})-\hat{y}(t_{i};\eta ),$ and the least squares loss is

$$L(\eta )=\sum_{i=1}^{n}{\left(y(t_{i})-\hat{y}(t_{i};\eta )\right)}^{2}.$$ (11)

then the model becomes $Y_{i}=Z_{i}(2-\eta )$. Thus the optimal η∈[1,2] minimizes


$$L(\eta )=\sum_{i=1}^{n}{\left(Y_{i}-Z_{i}(2-\eta )\right)}^{2}=\sum_{i=1}^{n}{\left(Z_{i}(\eta -1)-(Z_{i}-Y_{i})\right)}^{2}.$$

Taking the derivative with respect to *η* and solving the normal equation gives the closed-form solution:

$$\hat{\eta }=\frac{\sum_{i=1}^{n}Z_{i}(Z_{i}-Y_{i})}{\sum_{i=1}^{n}Z_{i}^{2}}$$ (12)

### 3.3 Classification by fractional order

Based on our expectations (extreme classes close to fractional orders 1 and 2) about the *η* values, we established three qualitative classes for growth kinetics:

- Minimal growth temperature: η>1.875 (should cover 8–20 ^∘^C):
- Optimal growth temperature: 1.125<η≤1.875 (e.g., 24–37 ^∘^C): fractional order dynamics
- Maximal growth temperature: η≤1.125 (e.g., 38–41 ^∘^C): 1-st order dynamics

This classification is consistent with biological expectations and shows low intra-group variance in most cases (see Table 6). Thus, the fitted parameter *η* effectively captures temperature-dependent growth dynamics and enables semi-automatic classification.

**Table 6:** Mean and variance of fractional order at different temperatures.

Temperature	$\hat{\eta }$	Variance of $\hat{\eta }$	Classifier
40 ^∘^C	1.1085	0.00198007	Maximal
39 ^∘^C	1.0983	0.00040461	Maximal
38 ^∘^C	1.1524	0.00124493	Optimal
37 ^∘^C	1.3162	0.00464144	Optimal
35 ^∘^C	1.2504	0.00193977	Optimal
34 ^∘^C	1.2151	0.00169195	Optimal
33 ^∘^C	1.2436	0.00060530	Optimal
30 ^∘^C	1.2768	0.00125684	Optimal
26 ^∘^C	1.2616	0.02890875	Optimal
24 ^∘^C	1.4223	0.03489408	Optimal
18 ^∘^C	1.4979	0.00286900	Optimal
16 ^∘^C	1.8776	0.02177452	Minimal
12 ^∘^C	1.8686	0.01674017	Minimal
10 ^∘^C	1.9522	0.00960596	Minimal

We fixed the shape parameter ν=3. This choice was motivated by empirical testing: lower values (e.g., ν=1 or ν=2) consistently resulted in poorer fits. We estimated four free parameters: the fractional order *η*, the nonlinearity parameter *α*, offset *c*, and asymptotic level *A*. We performed parameter estimation using the odeint() routine, i.e., it was solved numerically using an integrator from SciPy. It employs the LSODA algorithm, which automatically detects stiffness and switches between a non-stiff Adams method and a stiff backward differentiation formula. Given the nonlinear and potentially stiff dynamics of our model (especially due to the exponent ν=3), LSODA frequently engaged the stiff solver mode during numerical integration.

**Fig 2:**
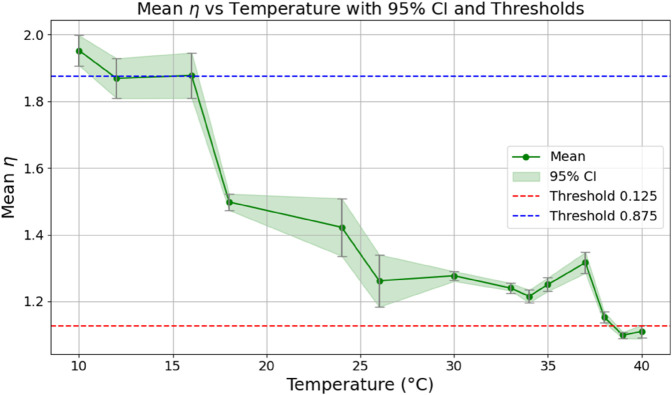
Mean values of the fitted fractional order *η* for biomass as a function of temperature, with vertical confidence intervals. Dashed horizontal lines represent classification thresholds of 1.125 and 1.875.

**Fig 3:**
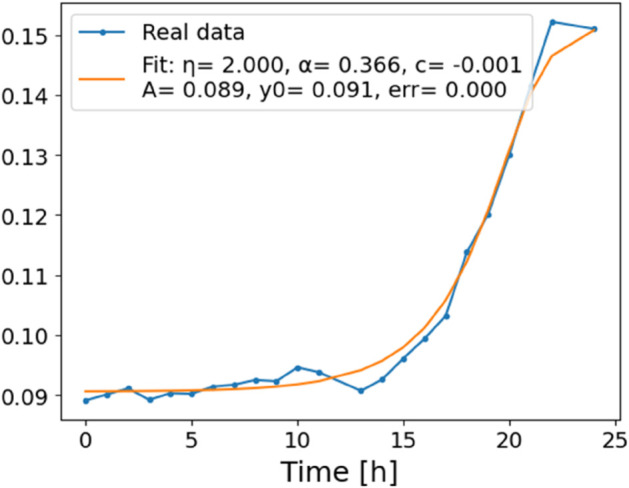
Estimation of parameters and fitting of proposed model, temperature 10 ^∘^C.

**Fig 4:**
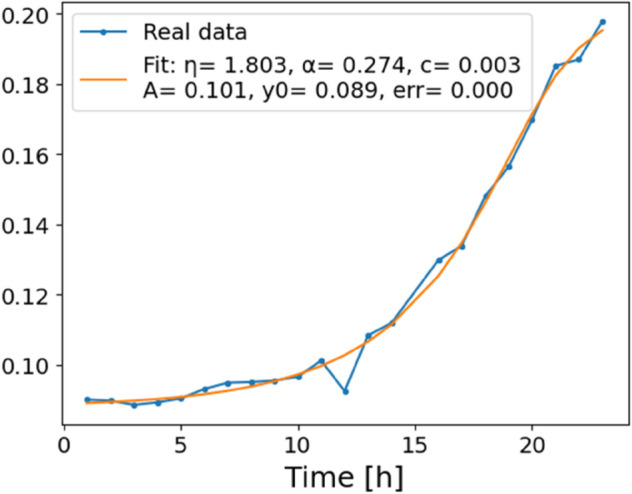
Estimation of parameters and fitting of proposed model, temperature 12 ^∘^C.

**Fig 5:**
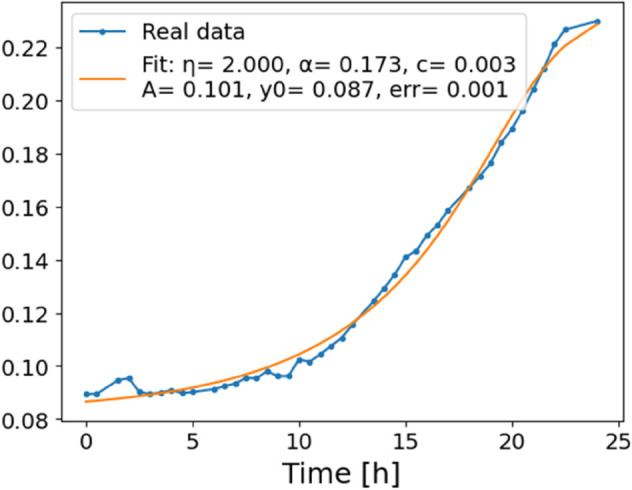
Estimation of parameters and fitting of proposed model, temperature 16 ^∘^C.

**Fig 6:**
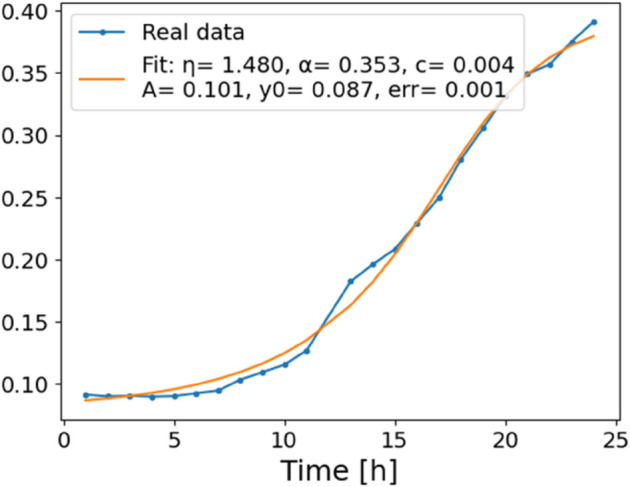
Estimation of parameters and fitting of proposed model, temperature 18 ^∘^C.

**Fig 7:**
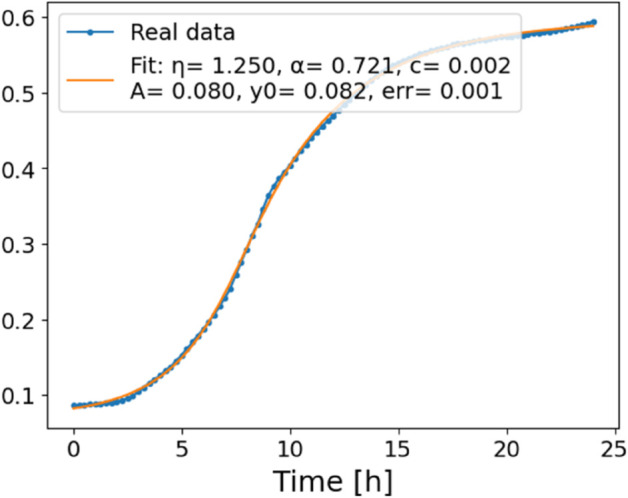
Estimation of parameters and fitting of proposed model, temperature 30 ^∘^C.

**Fig 8:**
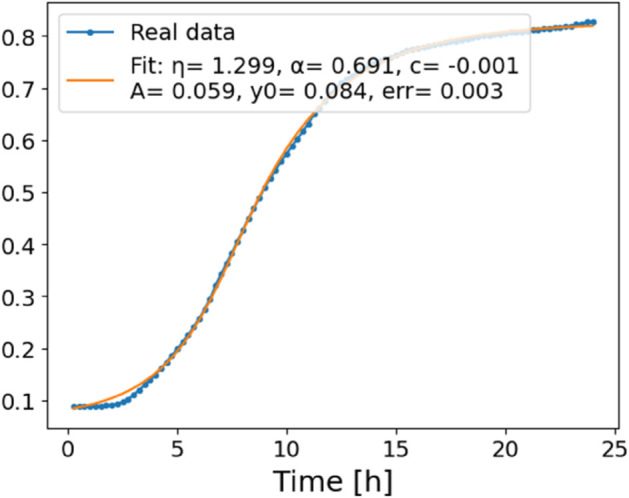
Estimation of parameters and fitting of proposed model, temperature 35 ^∘^C.

**Fig 9:**
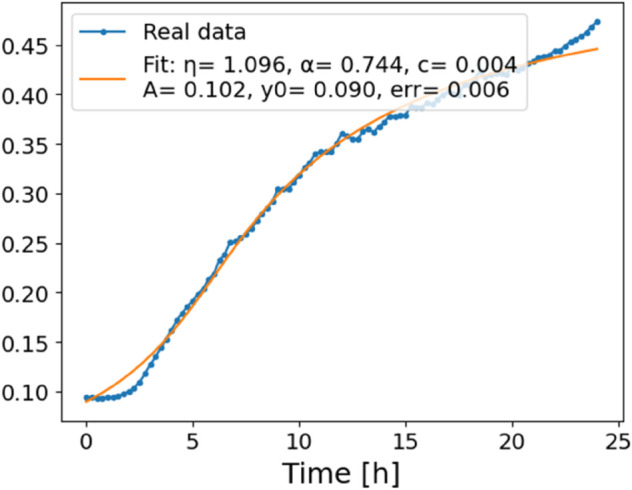
Estimation of parameters and fitting of proposed model, temperature 39 ^∘^C.

**Fig 10:**
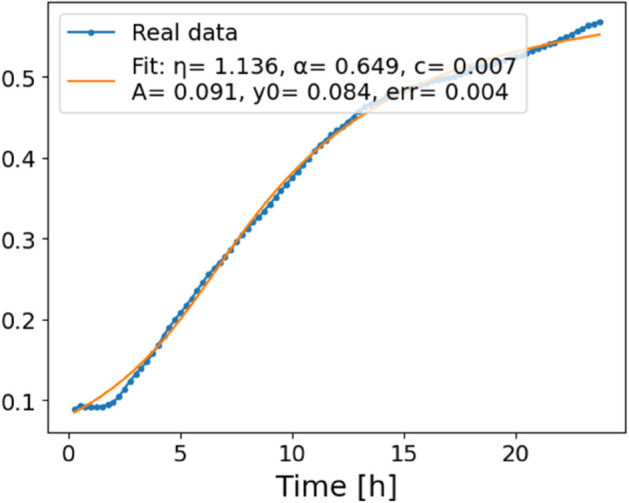
Estimation of parameters and fitting of proposed model, temperature 40 ^∘^C.

The estimated order *η* is given in Table 6. For example, for a temperature of 16 ^∘^C it is around 1.878, for 33 ^∘^C it is around 1.243, and for 39 ^∘^C it is around 1.098 (on average). The data reveal that the fractional order is strongly temperature-dependent. Lower temperatures (e.g., 10 ^∘^C–18 ^∘^C) correspond to higher *η* values (approaching 2), thus higher fractional orders, suggesting faster or more memory-driven dynamics and possibly accelerated or persistent growth behaviors. In contrast, at higher temperatures (30 ^∘^C–40 ^∘^C), the values of *η* are significantly lower, indicating dynamics closer to classical (1st order) behavior with diminished memory effects. This implies slower or more localized changes and slower growth processes. Overall, the observed trends highlight the critical role of temperature in modulating the fractional dynamics of *Cobetia marina* proliferation. See Fig 2 where data from Table 6 are plotted, and also refer to Figs 3-10 to view the fit. We have used normal-based confidence intervals with a coverage probability of 95%. We have also realized that the fractional order can drastically reduce time series autocorrelation. See Figs 42–49 in Appendix, where estimated *η*’s were used. This is significant in the case of a temperature of 16 ^∘^C. Stronger differentiation flattens the data more aggressively. It suppresses trends and autocorrelation, effectively reducing memory in the data. This removes long-term correlations between values.

**Fig 11:**
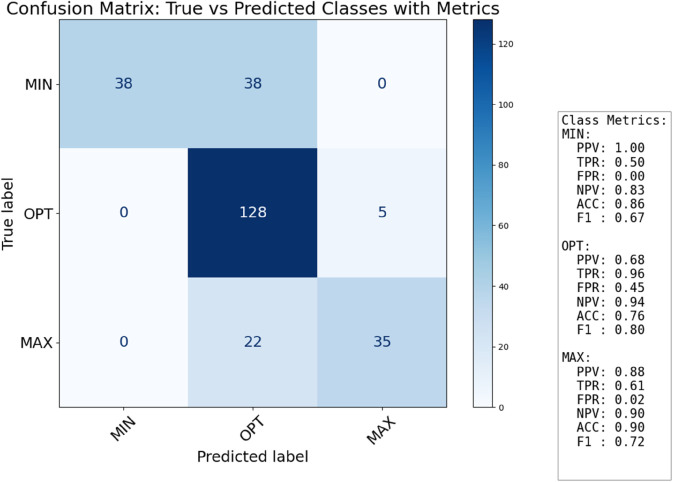
Confusion matrix for classification.

The confusion matrix for classification, presented in Fig 11, provides a comprehensive overview of our model’s performance in categorizing growth temperatures based on the estimated fractional order *η*. For the Minimal class, the model achieves perfect precision (PPV: 1.00), meaning all instances predicted as “Minimal" were indeed minimal growth temperatures. However, its recall (TPR: 0.50) indicates that only half of the actual minimal growth temperature cases were correctly identified, suggesting some true minimal cases were misclassified into other categories (though the specific matrix shows no misclassifications from the “True label MIN" row, implying the TPR 0.50 is an overall metric across a larger dataset). The False Positive Rate (FPR) for this class is 0.00, demonstrating that no instances from other classes were incorrectly labeled as Minimal. The Optimal class shows strong recall (TPR: 0.96), indicating that nearly all optimal growth temperature instances were correctly identified. However, its precision (PPV: 0.68) is moderate, and the False Positive Rate (FPR: 0.45) is relatively high, suggesting that a notable portion of non-optimal cases were incorrectly classified as “Optimal". This indicates a tendency for the model to over-predict the “Optimal" class. For the Maximal class, the model exhibits good precision (PPV: 0.88) and a low False Positive Rate (FPR: 0.04), indicating a high degree of confidence in its positive predictions for this class. The recall (TPR: 0.61) is moderate, meaning that while predictions for “Maximal" are generally accurate, a significant number of actual maximal growth temperature cases were not captured. Overall, the classification performance is robust, especially for the “Optimal" class’s recall and the “Minimal" and “Maximal" classes’ precision, validating the fractional-order approach for temperature-dependent growth classification.

### 3.4 Fractional classification and fitting models for growth kinetics of mutants of *Cobetia marina*

Following the same methodology as in the previous section, we applied the fractional-order model to mutant strains and estimated the order parameter *η* using the temperature-specific growth data. Mutant data often exhibit more variability or subtle shifts in growth dynamics compared to wild-type strains, which might be more robust or follow a clearer, more predictable pattern. For the mutants, we specifically used the integral form of the model to estimate *η*, unlike the differential model used for the wild-type. This approach was necessary because the variance of *η* was found to be too large when using the differential model for mutant data. The reason is that since the measurements are inherently noisy, taking numerical derivatives of such noisy data amplifies this noise considerably. Integral models inherently smooth out noise because integration is a smoothing operation. Instead of relying on noisy rates of change, the integral form works with accumulated values, which are generally more stable and less susceptible to measurement noise. Our aim was to assess whether the fractional classification scheme (based on thresholds η=1.125 and η=1.875) remains valid for mutant behavior and whether mutants preserve or deviate from wild-type trends.

As shown in Table 7, the mutants consistently show lower values of the fractional order *η* across the tested temperature range. For example, at 40–41 ^∘^C, the estimated values of *η* for mutants drop below 1.03, placing them well within the maximal temperature class. Meanwhile, for intermediate temperatures such as 30 ^∘^C–35 ^∘^C, the estimated *η* values (ranging between 1.19 and 1.24) still fall within the optimal class.

**Table 7:** Mean and variance of fractional order at different temperatures for mutants.

Temperature	$\hat{\eta }$	Variance of $\hat{\eta }$	Classifier
41 ^∘^C	1.027709338	0.004731089	Maximal
40 ^∘^C	1.010347714	0.000349916	Maximal
35 ^∘^C	1.211970365	0.013579725	Optimal
33 ^∘^C	1.23919907	0.010281213	Optimal
30 ^∘^C	1.194604411	0.017960449	Optimal
24 ^∘^C	1.196490098	0.01717118	Optimal

In Fig 12, we display the temperature dependence of *η* for mutants along with classification boundaries. The separation between classes is preserved, but with lower overall values, implying a compression of dynamic range. This pattern is further supported by the model fits (Figs 13–20, and Fig 21–24, which exhibit reduced curvature and smoother, slower transitions to saturation, in contrast to the wild-type fits shown in Fig 3–10. In the context of the fractional differential model, this corresponds to a smaller magnitude of the fractional growth rate, modeled by the CF derivative ${𝒟}^{\eta }y(t)$. Specifically, the mutant strains exhibit a lower maximum of this rate, approximated by


$$\max_{t}\left\{{𝒟}^{\eta }y(t)\right\}\approx \max_{t}\left\{\alpha (y(t)-A)\left(1-{\left(\frac{y(t)-A}{K-A}\right)}^{\nu }\right)\right\},$$

where the right-hand side corresponds to the nonlinear growth function $\tilde{G}(y)$. The reduced magnitude and flatter shape of this term across time for the mutant strains reflect both weaker dynamic responsiveness and reduced memory effects—consistent with lower fitted values of the fractional order $\hat{\eta }$. These effects are visualized in Fig 2 (based on Table 6), and compared to the wild-type reference dynamics shown in Figs 3–10.

**Fig 12:**
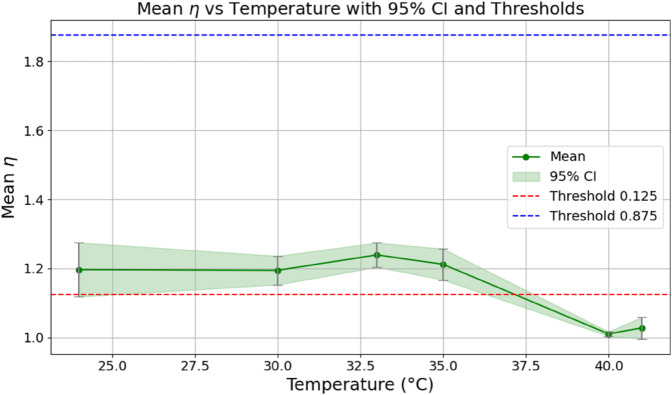
Mean values of the fitted fractional order *η* as a function of temperature, with vertical confidence intervals. Dashed horizontal lines represent classification thresholds of 1.125 and 1.875.

**Fig 13:**
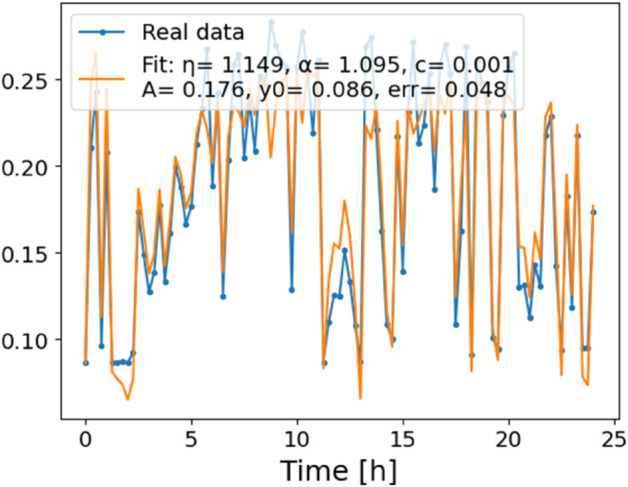
Estimation of parameters and fitting of the proposed model for mutants, temperature 24 ^∘^C.

**Fig 14:**
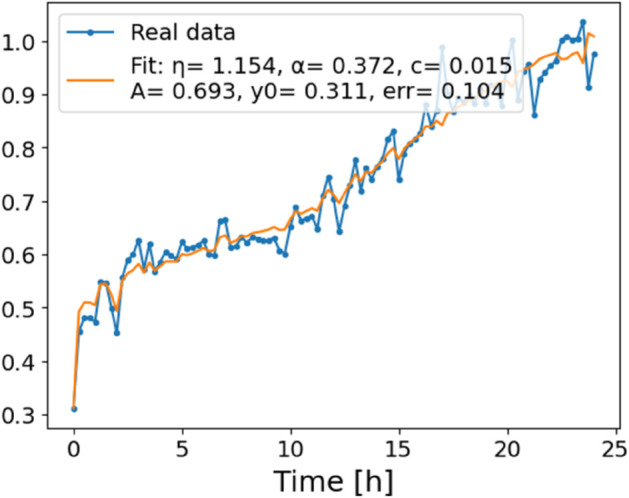
Estimation of parameters and fitting of the proposed model for mutants, temperature 24 ^∘^C.

**Fig 15:**
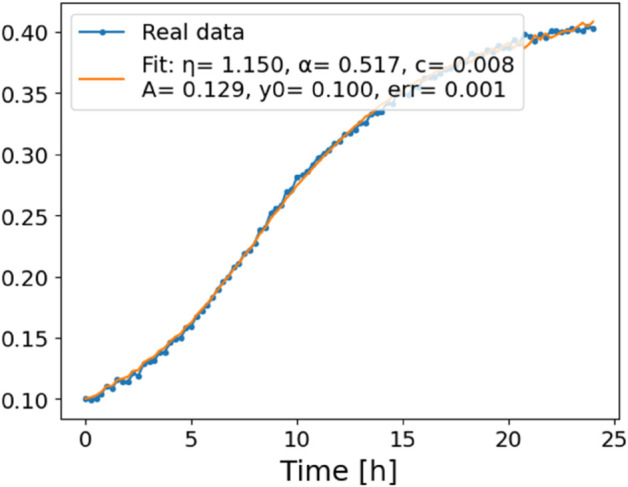
Estimation of parameters and fitting of the proposed model for mutants, temperature 30 ^∘^C.

**Fig 16:**
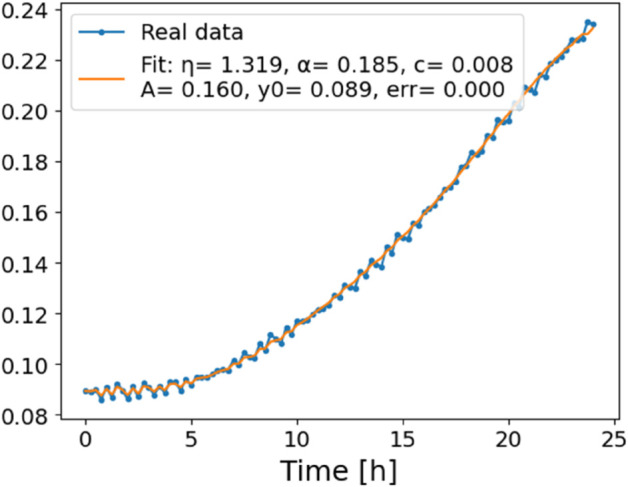
Estimation of parameters and fitting of the proposed model for mutants, temperature 30 ^∘^C.

**Fig 17:**
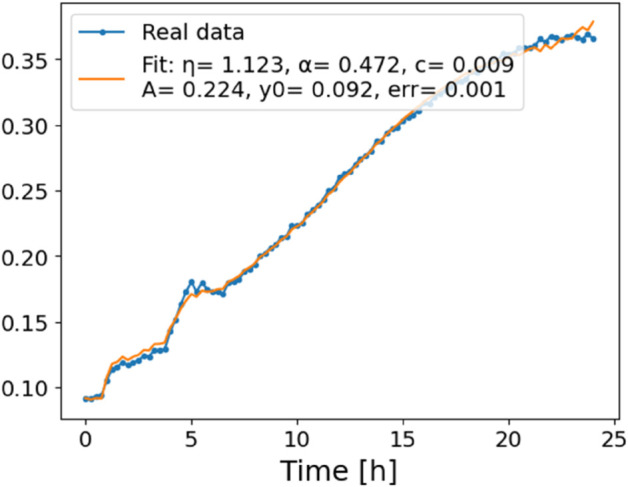
Estimation of parameters and fitting of the proposed model for mutants, temperature 33 ^∘^C.

**Fig 18:**
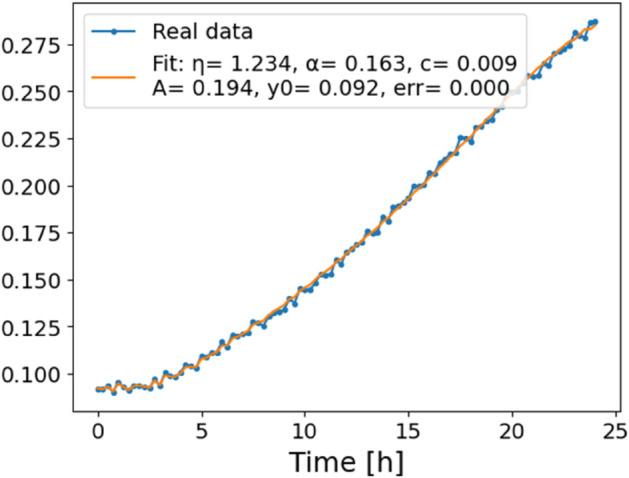
Estimation of parameters and fitting of the proposed model for mutants, temperature 33 ^∘^C.

**Fig 19:**
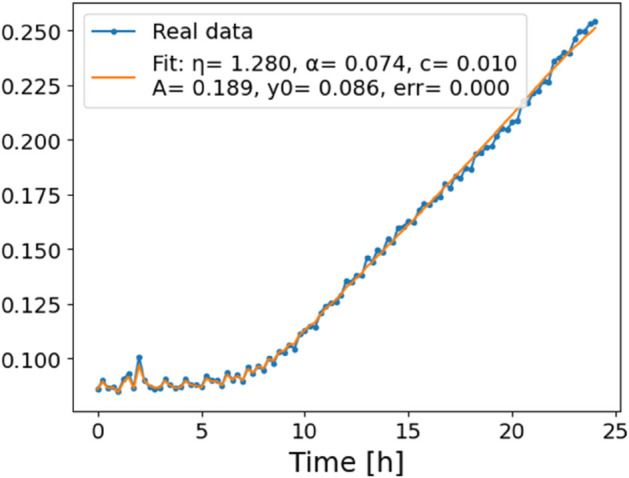
Estimation of parameters and fitting of the proposed model for mutants, temperature 35 ^∘^C.

**Fig 20:**
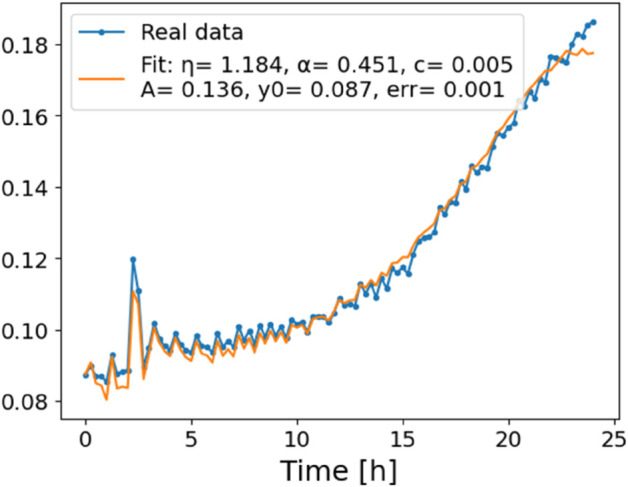
Estimation of parameters and fitting of the proposed model for mutants, temperature 35 ^∘^C.

**Fig 21:**
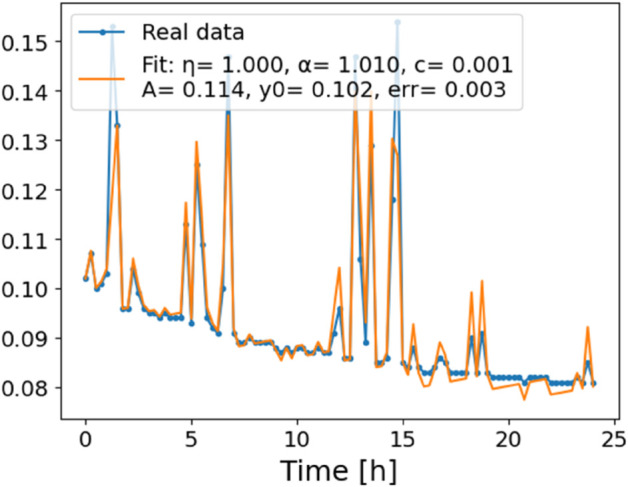
Estimation of parameters and fitting of the proposed model for mutants, temperature 40 ^∘^C.

**Fig 22:**
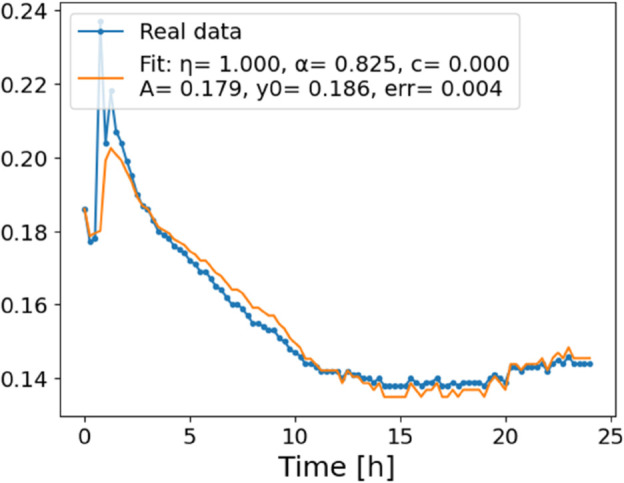
Estimation of parameters and fitting of the proposed model for mutants, temperature 40 ^∘^C.

**Fig 23:**
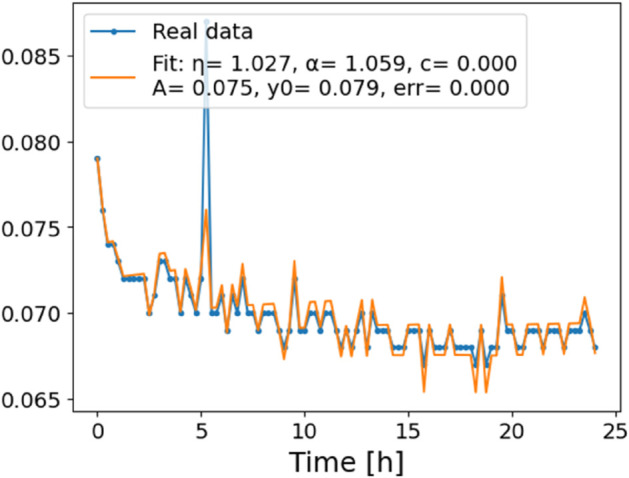
Estimation of parameters and fitting of the proposed model for mutants, temperature 41 ^∘^C.

**Fig 24:**
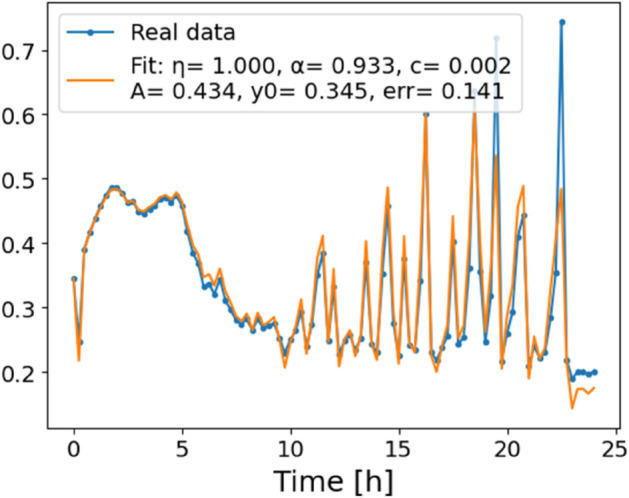
Estimation of parameters and fitting of the proposed model for mutants, temperature 41 ^∘^C.

**Fig 25:**
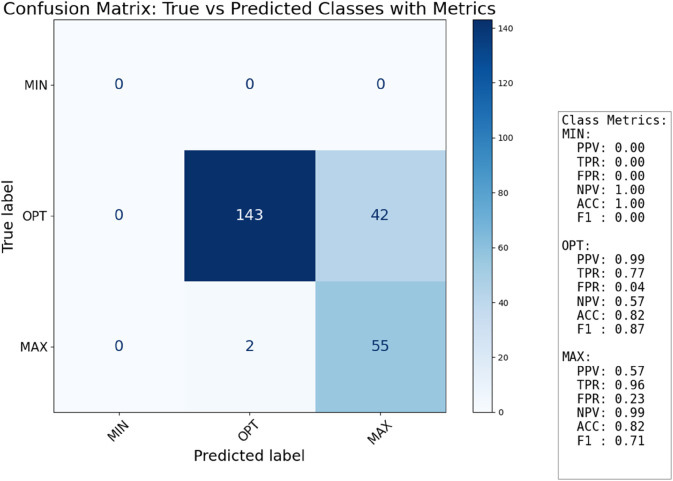
Confusion matrix for classification for kinetics growth of Cobetia marina mutants.

The confusion matrix for the mutant classification, displayed in Fig 25, reveals distinct patterns in classification performance compared to the wild-type. A critical observation is the complete failure to classify the Minimal growth temperature class. Both the precision (PPV: 0.00) and recall (TPR: 0.00) for this class are zero, and the confusion matrix shows no instances predicted as “MIN". This suggests that either no true “Minimal" temperature mutant samples were present in the test set that contributed to these metrics, or, more likely, that the estimated *η* values for mutant strains at lower temperatures consistently fall outside the defined η>1.875 threshold for the “Minimal" class, pushing them into “Optimal" or “Maximal" categories. This could imply a fundamental shift in the fractional dynamics of mutants at traditionally minimal growth temperatures. In contrast, the classification of the Optimal class for mutants is highly successful, boasting exceptionally high precision (PPV: 0.99) and good recall (TPR: 0.77). The False Positive Rate (FPR: 0.04) is also very low, indicating that the model is highly accurate when predicting an optimal growth temperature for mutants. For the Maximal class, the model achieves excellent recall (TPR: 0.96), meaning almost all true maximal growth temperature instances were correctly identified. However, its precision (PPV: 0.57) is notably lower than for the “Optimal" class, and the False Positive Rate (FPR: 0.23) is higher. This suggests that while the model effectively captures most “Maximal" cases, it also frequently misclassifies other growth conditions (likely “Optimal") as “Maximal". This indicates a potential broadening of the “Maximal" prediction window for mutants. These results highlight that while the fractional classification scheme remains generally applicable to mutants, the performance for specific classes, particularly “Minimal," is significantly altered, reflecting the stationary phase bacterial growth profile.

### 3.5 Neural network using SPOCU classifier

As shown in Table 8, SPOCU outperformed ReLU in most configurations, particularly when combined with MAE, Log-Cosh, and MSLE loss functions, whereas ReLU showed slightly better results only under MSE and MAPE conditions.

**Table 8:** Comparisons.

Optimizer-L.F	SPOCU	ReLU
Adam-MSE	×	✓
Adam-MAE	✓	×
Adam-LogCosh	✓	×
Adam-MAPE	!	!
Adam-MSLE	✓	×
SGD-MSE	✓	×
SGD-MAE	✓	×
SGD-LogCosh	✓	×
SGD-MAPE	×	✓
SGD-MSLE	!	!

#### 3.5.1 Temperature classification Using SPOCU classifier.

The results of the three architectures in the binary classification task were compared using the accuracy metric for the classification of biofilm production. The results showed that, while the ReLU and SELU activation functions achieved accuracies of 0.9908 and 0.9816, respectively, the use of SPOCU led to a significantly higher accuracy of 0.9931. In the three-class classification task, ReLU and SELU achieved accuracies of 0.9931 and 0.9885, respectively, while SPOCU again outperformed both with an accuracy of 0.9954. This indicates that the SPOCU activation function is more effective in distinguishing between high and low biofilm production in this context.

**Fig 26:**
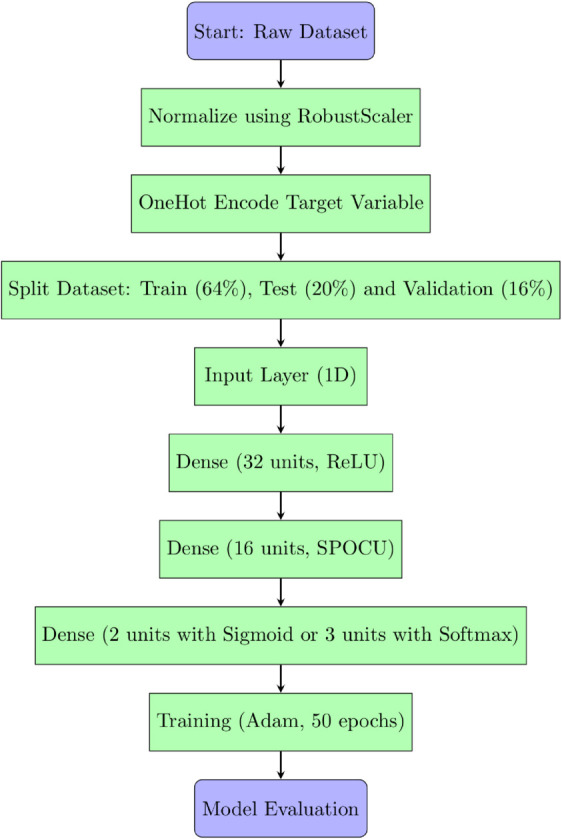
Work flow chart for SPOCU-based neural network.

This study demonstrated that it is possible to effectively classify biofilm production in *Cobetia marina* using neural network models and data from growth kinetics. Differences in activation functions have a direct impact on model performance, and the use of the SPOCU activation function with tuned parameters showed promising results compared to ReLU and SELU. The SPOCU function achieved the best performance, with an accuracy of 0.6, making it the most suitable choice for this classification task. The workflow chart for SPOCU-based Neural Network is plotted in Fig 26 and in the next list.

- **Raw Dataset**: Initial data collection. We used growth (OD600) and biofilm (OD540) data of *Cobetia marina*. Preprocessing is necessary to handle noise, missing values, and outliers before model input.
- RobustScaler: transforms the feature values to reduce the impact of outliers. Unlike MinMax or StandardScaler, it uses the median and interquartile range (ideal for datasets with skewed distributions or extreme values). It corrects variability across replicates and temperatures.
- **OneHot Encode Target Variable**: converts categorical labels into a binary matrix for classification tasks, encoding biological growth classes for training.
- **Split Dataset**: the training set for fitting the model and learning patterns, the validation set for tuning hyperparameters and preventing overfitting, the test set for evaluating the model’s generalization performance on unseen data. This ensures reproducible analysis across biological replicates.
- **The neural network’s input layer is 1D** indicates the data is in a flat vector format (e.g., time series) rather than images or sequences, representing the biofilm-to-biomass signal for each replicate.
- **Dense Layer (32 units, ReLU):** is fully connected with 32 neurons and ReLU activation, introducing non-linearity, allowing the network to learn complex patterns. It captures patterns in growth curve variability.
- **Dense Layer (16 units, SPOCU):** utilizes the SPOCU activation function, which enhances learning dynamics with good properties. It aids in capturing intricate data relationships, improving stability, and distinguishing subtle differences in growth and biofilm formation.
- **Dense Layer (2 units with Sigmoid or 3 units with Softmax):** Sigmoid is used for binary classification, outputs probabilities between 0 and 1. Softmax is used for multi-class classification. The number of units corresponds to the number of target classes. This classifies samples into Minimal, Optimal, or Maximal biofilm production
- **Training (Adam, 50 epochs):** The model is trained with the Adam optimizer over 50 epochs to refine its weights and learn biological patterns in the data.
- **Model Evaluation:** After training, the model is evaluated using metrics like accuracy, precision, recall, F1-score, or loss to determine its performance and readiness for deployment or further tuning. This step validates the classification of *Cobetia marina* growth categories.

#### 3.5.2 Model for biofilm inhibitors and mutants.

First, Table 9 shows the general performance metrics for each tested model when a biofilm inhibitor is used at different concentrations. Tables 10 and 11 show the performance metrics in the models in binary or three-class classification on mutants analysis.

**Table 9:** General metrics of the tested models on inhibitor analysis.

Model	Accuracy	Precision	Recall	F1-Score
SPOCU	0.6477	0.5870	0.9350	0.7257
ReLU	0.5568	0.5345	0.7209	0.6139
SeLU	0.5568	0.5345	0.7209	0.6139

**Table 10:** General metrics of the tested models on binary mutants analysis.

Model	Accuracy	Precision	Recall	F1-Score
SPOCU	1	1	1	1
ReLU	1	1	1	1
SeLU	1	1	1	1

**Table 11:** General metrics of the tested models on three-class mutants analysis.

Model	Accuracy	Precision	Recall	F1-Score
SPOCU	0.9818	0.9885	0.9792	0.9834
ReLU	1	1	1	1
SeLU	1	1	1	1

#### 3.5.3 Comparing transfer functions: ReLU, SELU, SPOCU.

**Fig 27:**
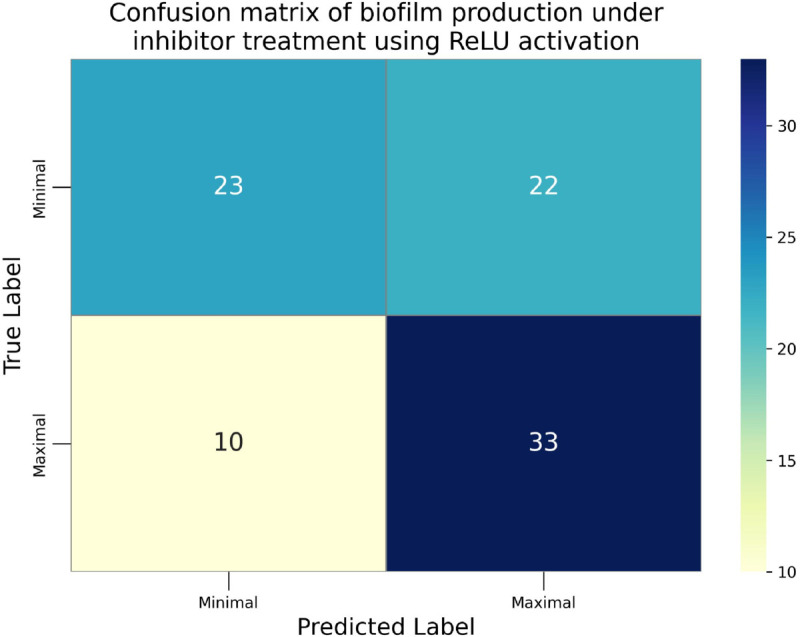
Confusion matrix of the models on inhibitor analysis, ReLu.

**Fig 28:**
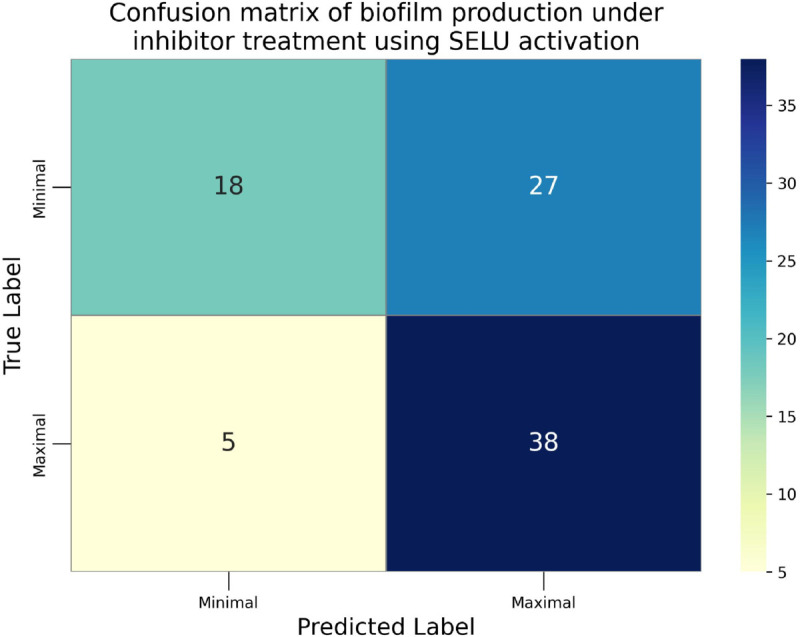
Confusion matrix of the models on inhibitor analysis, SELU.

**Fig 29:**
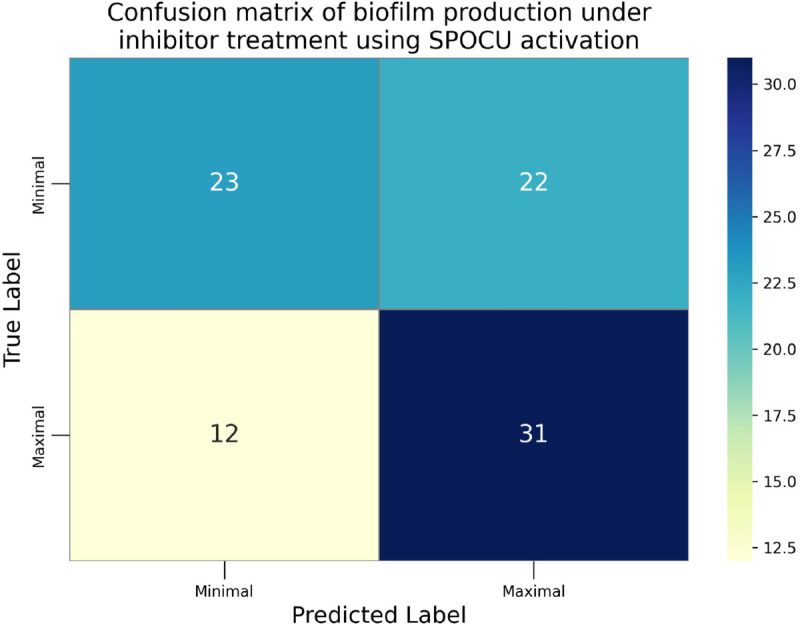
Confusion matrix of the models on inhibitor analysis, SPOCU.

**Fig 30:**
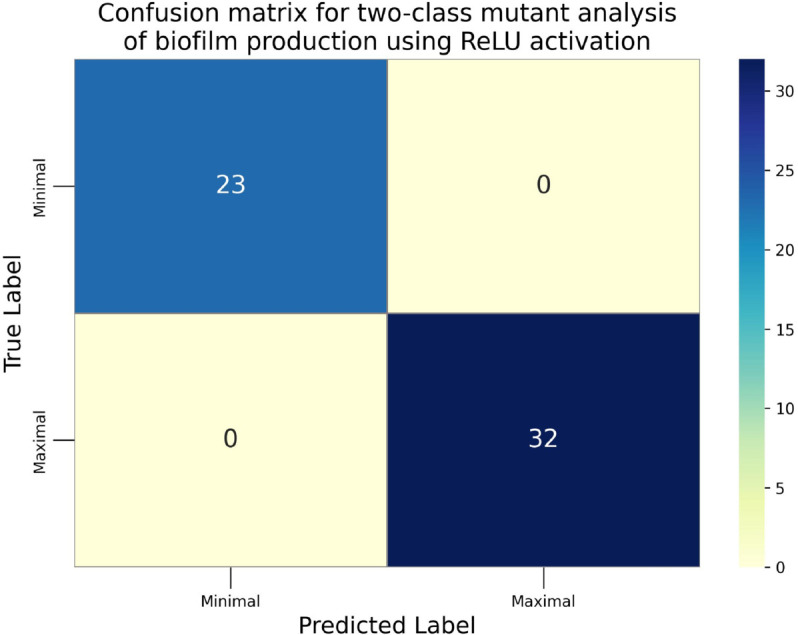
Confusion matrix of the models on binary mutant analysis, ReLu.

**Fig 31:**
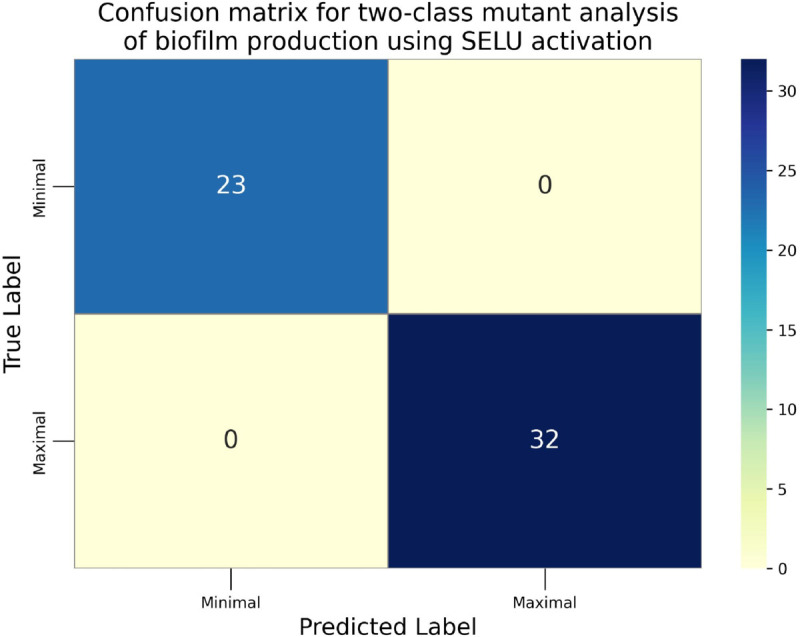
Confusion matrix of the models on binary mutant analysis, SELU.

**Fig 32:**
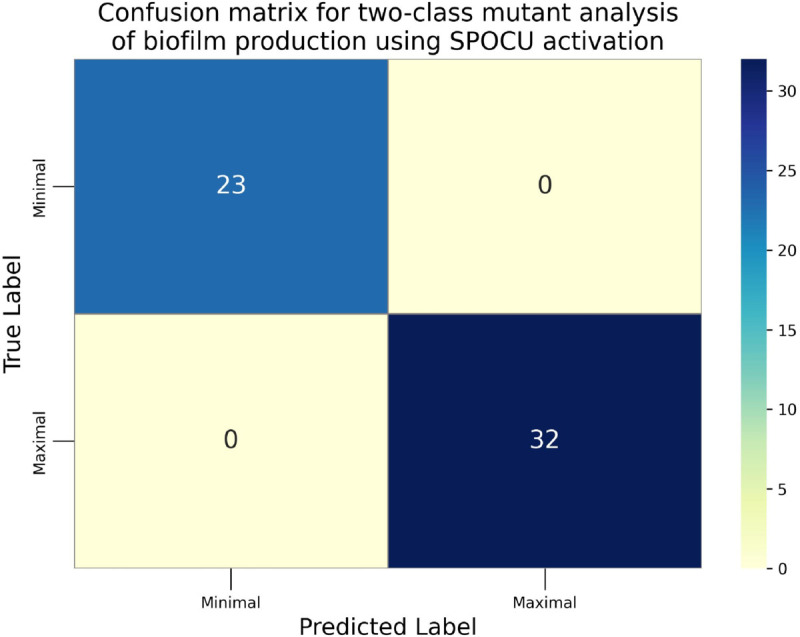
Confusion matrix of the models on binary mutant analysis, SPOCU.

**Fig 33:**
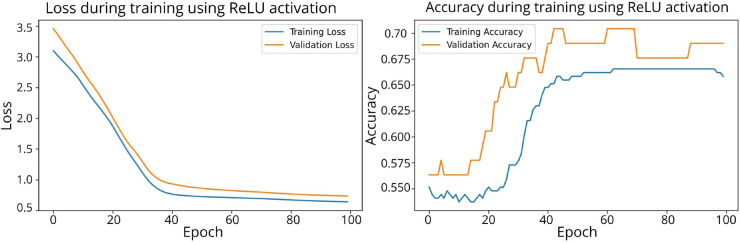
Loss and accuracy per epoch during training on inhibitor analysis, ReLu.

**Fig 34:**
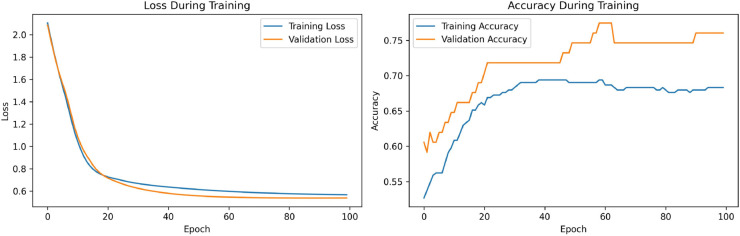
Loss and accuracy per epoch during training on inhibitor analysis, SELU.

**Fig 35:**
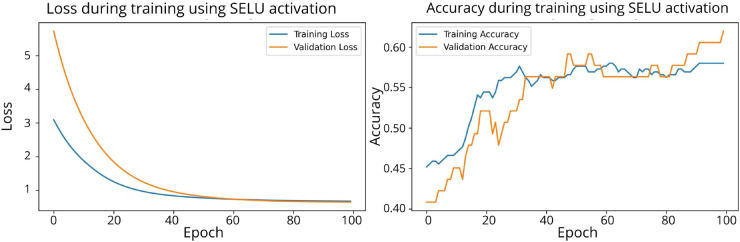
Loss and accuracy per epoch during training on inhibitor analysis, SPOCU.

**Fig 36:**
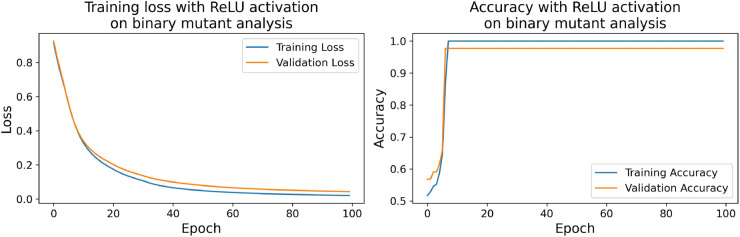
Loss and accuracy per epoch during training on binary mutant analysis, ReLu.

**Fig 37:**
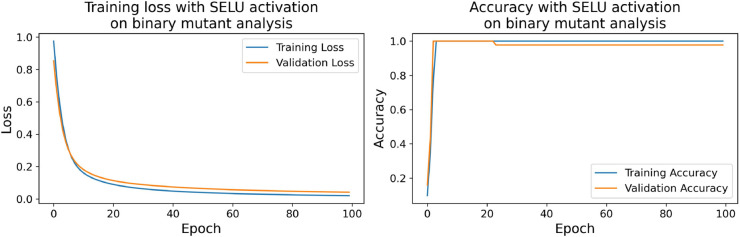
Loss and accuracy per epoch during training on binary mutant analysis, SELU.

**Fig 38:**
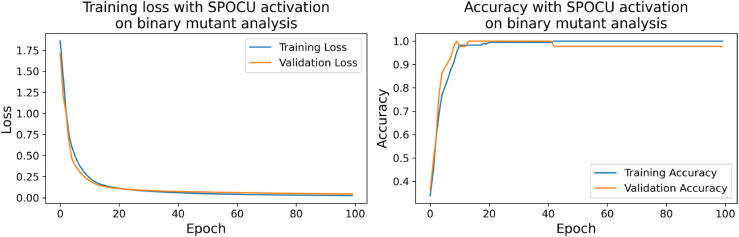
Loss and accuracy per epoch during training on binary mutant analysis, SPOCU.

**Fig 39:**
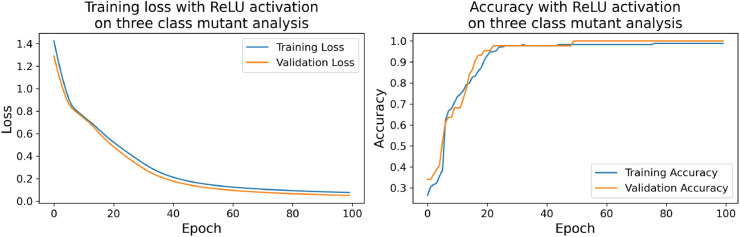
Loss and accuracy per epoch during training on three-class mutant analysis, ReLu.

**Fig 40:**
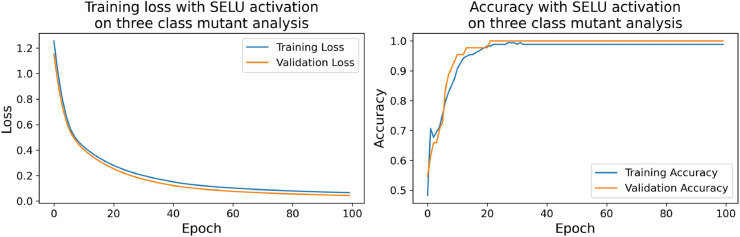
Loss and accuracy per epoch during training on three-class mutant analysis, SELU.

**Fig 41:**
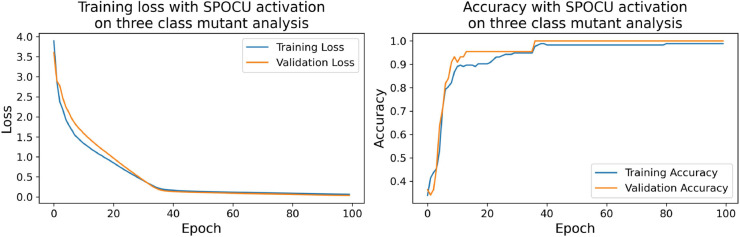
Loss and accuracy per epoch during training on three-class mutant analysis, SPOCU.

The confusion matrices for the inhibitor concentration analysis and the binary mutants analysis are presented in Figs 27–32, respectively. Furthermore, Figs 33–41 illustrate the training process across 100 epochs for the three distinct models developed for biofilm production analysis using the inhibitor concentration approach, the two-class classification of mutants, and the three-class classification of mutants, respectively. In addition, the specific performance metrics for each of these analyses are detailed in Tables 12–14. They summarize classification metrics across activation functions. Under inhibitor conditions (Table 12), SPOCU outperformed ReLU and SELU, while in binary and three-class mutant analyses (Tables 13 and14) all models achieved near-perfect accuracy, with SPOCU showing a slight edge. In Figs 33—38, validation accuracy occasionally exceeds training accuracy. This transient effect may arise from lower noise or complexity in the validation subset, as well as the stochastic nature of training combined with normalization and regularization, which can penalize training performance while enhancing generalization. Additionally, random partitioning of biologically variable replicates may yield subsets of differing difficulty. Both curves ultimately converge to similar high-accuracy levels, supporting the robustness of our findings.

**Table 12:** Combined classification metrics across activation functions on inhibitor analysis.

Activation	Metric	Precision	Recall	F1-score	Support
ReLU	Class: Minimal	0.600	0.400	0.480	45
	Class: Maximal	0.534	0.721	0.614	43
	Accuracy	0.557	0.557	0.557	0.557
	Macro avg	0.567	0.560	0.547	88
	Weighted avg	0.568	0.557	0.545	88
SELU	Class: Minimal	0.600	0.400	0.480	45
	Maximal	0.534	0.721	0.614	43
	Class: Accuracy	0.557	0.557	0.557	0.557
	Macro avg	0.567	0.560	0.547	88
	Weighted avg	0.568	0.557	0.545	88
SPOCU	Class: Minimal	0.889	0.356	0.508	45
	Class: Maximal	0.586	0.953	0.726	43
	Accuracy	0.648	0.648	0.648	0.648
	Macro avg	0.737	0.655	0.617	88
	Weighted avg	0.741	0.648	0.614	88

**Table 13:** Combined classification metrics across activation functions on binary mutants analysis.

Activation	Metric	Precision	Recall	F1-score	Support
ReLU	Class: Minimal	1	1	1	23
	Class: Maximal	1	1	1	32
	Accuracy	1	1	1	1
	Macro avg	1	1	1	55
	Weighted avg	1	1	1	55
SELU	Class: Minimal	1	1	1	23
	Class: Maximal	1	1	1	32
	Accuracy	1	1	1	1
	Macro avg	1	1	1	55
	Weighted avg	1	1	1	55
SPOCU	Class: Minimal	1	1	1	23
	Class: Maximal	1	1	1	32
	Accuracy	1	1	1	1
	Macro avg	1	1	1	55
	Weighted avg	1	1	1	55

**Table 14:** Combined classification metrics across activation functions on three-class mutants analysis.

Activation	Metric	Precision	Recall	F1-score	Support
ReLU	Class: Minimal	1	1	1	11
	Class: Optimal	1	1	1	16
	Class: Maximal	1	1	1	28
	Micro avg	1	1	1	55
	Macro avg	1	1	1	55
	Weighted avg	1	1	1	55
SELU	Class: Minimal	1	1	1	11
	Class: Optimal	1	1	1	16
	Class: Maximal	1	1	1	28
	Micro avg	1	1	1	55
	Macro avg	1	1	1	55
	Weighted avg	1	1	1	55
SPOCU	Class: Minimal	1	1	1	11
	Class: Optimal	1	0.938	0.968	16
	Class: Maximal	0.966	1	0.982	28
	Micro avg	0.982	0.982	0.982	55
	Macro avg	0.989	0.979	0.983	55
	Weighted avg	0.982	0.982	0.982	55

## 4 Discussion

### 4.1 Fractional-derivative-based classification

The kinetics of microbial growth have been extensively studied to understand growth phases and to model and optimize growth based on biomass or metabolite production variables. This research aims to generate mathematical and statistical models. In most cases, the goal has been to model under optimized laboratory conditions, aiming for low data dispersion generated by experimental designs, procedures, equipment, and uncontrolled biological factors. In the present study, *Cobetia marina* was used as a model organism capable of growing over a wide range of temperatures. Growth kinetics were differentiated by temperatures (minimum, optimal, and maximum) and showed high data dispersion when the growth temperature deviated from the optimal range (33-35 ^∘^C). This scenario proved suitable for generating data that reflect the biological complexity associated with the remarkable adaptive capacity of *Cobetia marina* to grow at different temperatures and to generate data analysis models of growth kinetics phenotype in response to temperature.

From a classification standpoint, the mutant data fit cleanly into the optimal and maximal categories using the previously established thresholds. This validates the robustness of the fractional classification rule while revealing genotype-dependent shifts in dynamic behavior. Overall, these results confirm that the fractional-order framework remains a valid and interpretable tool even under genetic perturbations.

Fractional differentiation acts like a “memory eraser": the closer its order is to 1 (or higher), the more it removes low-frequency (long-memory) components. We are almost/essentially applying a second derivative, which emphasizes very local (instantaneous) changes and eliminates correlation over time. Based on this interpretation, we expected that 38 ^∘^C and above would belong to the High class (low *η*). However, empirical results showed that 38 ^∘^C fell within the Medium class, suggesting that this temperature represents a transitional regime between second and first-order dynamics. This deviation highlights the nuanced biological response near thermal limits and confirms that *η* captures more than just the growth rate; it reflects a deeper structural property of the system’s response dynamics. Among the fitted parameters, only *η* demonstrated meaningful variation correlated with temperature. Other parameters acted primarily as local curve-shaping factors and were not informative for classification.

In bacteria, there are examples of organisms capable of growing over a wide range of temperatures, known as eurytherms, and others that grow within a very narrow range of temperatures, known as stenotherms. Both cases are examples of evolutionary strategies, either specialization (stenothermy) or generalism (eurythermy), which are associated with the ability to colonize and inhabit a specific ecosystem. With a minimum modelable growth temperature of 10 ^∘^C, an optimal of 35 ^∘^C, and a maximum of 41 ^∘^C, *Cobetia marina*, the model organism of this study, can be defined as having a generalist strategy and thus as a mesophilic eurythermal bacterium. This confers a competitive fitness with the ability to colonize and inhabit different habitats within the marine ecosystem. However, the original data show that at different temperatures, *Cobetia marina* exhibits different performances in terms of growth (biomass formation and growth rate), allowing for the establishment of different models and classification systems presented in this manuscript. Both eurythermic and stenothermic organisms employ molecular, metabolic, physiological, and ecological mechanisms to drive the final biological fitness in an ecosystem. We can summarize the obtained results as follows:

- At lower temperatures ranging 10 ^∘^C to 16 ^∘^C   the fitted fractional derivative order *η* tends to be close to the integer value 2. This suggests behavior resembling second-order systems. A fractional order close to an integer can significantly reduce or even eliminate the memory effect. Fractional-order systems, unlike their integer-order counterparts, possess a memory effect, meaning their behavior is influenced by past events. As the fractional order approaches an integer value, the memory effect weakens, and when it reaches an integer value, the memory effect is typically eliminated. The memory effect could be a reflection of physiology and metabolism resulting from specific gene expression regulation circuits. The activation of regulatory proteins triggers metabolisms associated with structural membrane changes and the accumulation of reserve material, leading to biofilm formation and reducing growth in relation to biomass and growth rate.
- At higher temperatures ranging 38 ^∘^C to 40 ^∘^C the fitted order is closer to 1, i.e., *η* near one, which corresponds to first-order dynamics. This could be associated with thermal stress metabolism in bacteria, and in the case of *Cobetia marina*, a high growth rate and low resulting biomass are observed.
- Mid-range temperatures ranging 24 ^∘^C to 33 ^∘^C correspond to intermediate *η* values with optimal growth observed around η≈1.25. This represents a memory effect where there is a balance with the reactivity effect, and it can be associated with the range in which *Cobetia marina* finds its optimal growth temperature, with the highest growth rate and greater biomass formed. In this condition of balance between memory and reactivity, *Cobetia marina* could be deploying regulatory circuits aimed at maximizing the use of nutrients to enhance its growth and dissemination.

With the aim of generating conditions for phenotypic diversity in growth kinetics of the study model *Cobetia marina*, random mutants were obtained, and their growth curves were determined and classified using the models developed in this study for testing and validation. From a classification standpoint, the mutant data fit cleanly into the optimal and maximal categories using the previously established thresholds. This validates the robustness of the fractional classification rule while revealing genotype-dependent shifts in dynamic behavior. Overall, these results confirm that the fractional-order framework remains a valid and interpretable tool even under genetic perturbations.

### 4.2 Neural network using SPOCU Classifier for biofilm and growth kinetics modeling and prediction

Considering that the formation of bacterial biofilms is a relevant phenotype in various fields, the evaluation of inhibitory or enhancing agents is an important activity. In this study, artificial intelligence and mathematical and statistical tools were applied to analyze and classify complex data derived from more realistic conditions associated with the study model. For this purpose, *Cobetia marina* was exposed to a solution that inhibits growth and biofilm formation at different temperatures.

A feasible SPOCU implementation can guarantee the Self-Normalizing Network (SNN) condition. To the best of our knowledge, SPOCU is the most flexible transfer function that has the SNN property. SPOCU has been and continues to be tested in different cases and topics, evaluating its performance on problem-solving applications of machine learning, showing reasonable improvements over standard transfer functions, as seen in references such as [20] and [21]. In addition to these experiments, the effectiveness of SPOCU has been highlighted in previous studies. For instance, in [22], SPOCU was applied with parameters *c* = 1 and c=∞ in several artificial neural network models. The study concluded that SPOCU enhanced both robustness and prediction accuracy when tested for biofilms and kinetics data from *Cobetia marina*, especially in NARX and Elman networks, outperforming traditional functions like tanh. Similarly, in [23], SPOCU was evaluated in a 3-layer Extreme Learning Machine (ELM). The superior performance of SPOCU-based networks compared to conventional activation functions highlights the importance of selecting appropriate mathematical tools for biological applications.

## 5 Conclusion

Biological complexity and data analysis are ongoing challenges in biology. Studying realistic conditions can provide new insights into biological complexity and its modeling counterparts. Biofilms, microbial structures with various impacts, are a relevant area of study. In this work, we used the bacterial model *Cobetia marina* to study growth kinetics and biofilm formation. The integration of fractional derivative modeling with deep learning approaches provides a robust framework for understanding temperature-dependent bacterial behavior. This work demonstrates that fractional-order models, particularly those based on the CF derivative, offer a robust and biologically interpretable framework for modeling bacterial growth and biofilm formation under temperature stress. We conducted experiments at different temperatures and with a biofilm inhibitor. Our study focused on generating diverse data outputs and developing classifiers to determine bacterial growth temperatures and predict biofilm production. We studied three types of classifiers, where the fractional classifier has enjoyed good properties of flexibility in fractional approaches to growth models. We found that a neural network with the SPOCU transfer function outperformed other classifiers in predicting biofilm production for *Cobetia marina* and its mutants. Notice that the fractional classifiers offered interpretability and insight into dynamic behavior, while the SPOCU-based neural networks provided scalable, high-performance classification suitable for large and complex datasets. This methodological diversity strengthens the validity of our conclusions and showcases the importance of hybrid strategies when confronting biological complexity. The fractional order parameter *η* not only provides improved fits to experimental data but also serves as a proxy for biological memory and environmental responsiveness. Importantly, the sensitivity of *η* to underlying dynamic changes suggests that fractional models could be used as early-warning tools to detect anomalies or transitions in growth behavior. For instance, abrupt drops in *η* could indicate stress responses, metabolic shifts, or impending biofilm collapse. These features could be embedded into real-time monitoring systems for environmental microbiology, industrial bioreactors, or clinical diagnostics. This research demonstrates the power of integrating experimental microbiology with mathematical modeling and computational learning. By linking biological variability with fractional dynamics and machine learning, it contributes to a deeper understanding of life systems and offers a reproducible path toward modeling adaptive biological behavior under environmental stress.

## Appendix

**Fig 42:**
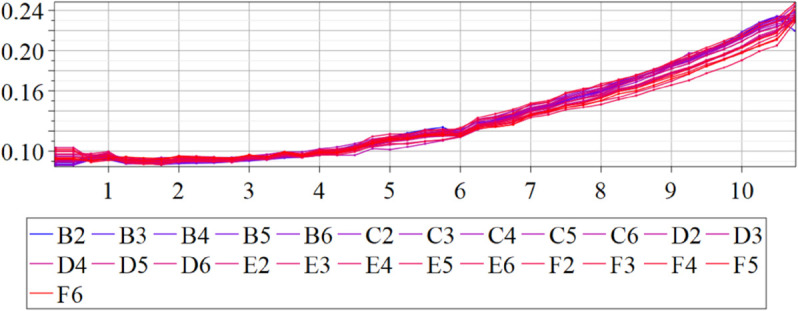
Original data, 16 ^∘^C.

**Fig 43:**
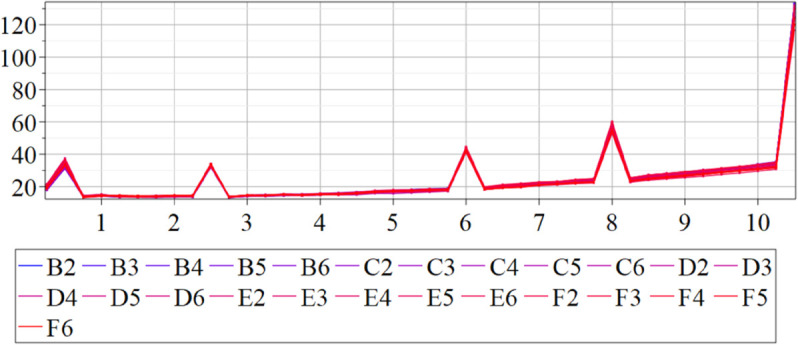
Fractional derivative with $\hat{\eta }$ = 1.877, 16 ^∘^C.

**Fig 44:**
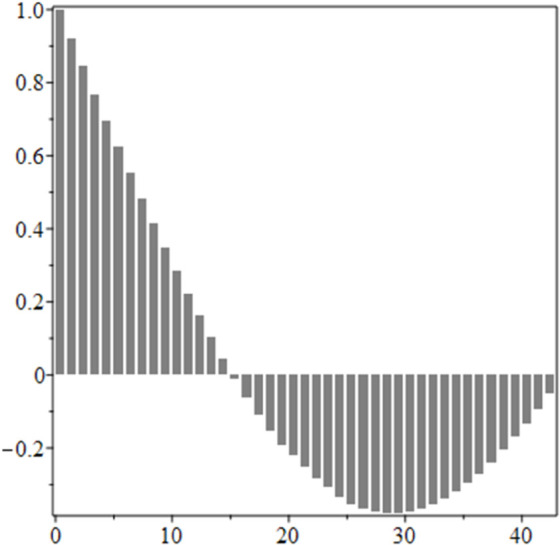
Autocorrelation of mean, 16 ^∘^C.

**Fig 45:**
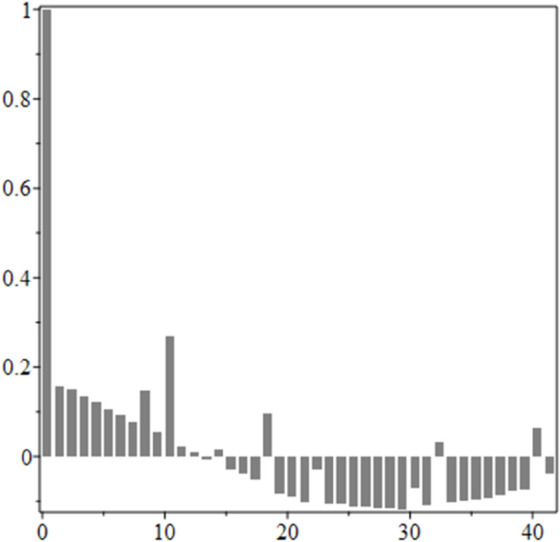
Autocorrelation of mean for fractional derivative with $\hat{\eta }$ = 1.877, 16 ^∘^C.

**Fig 46:**
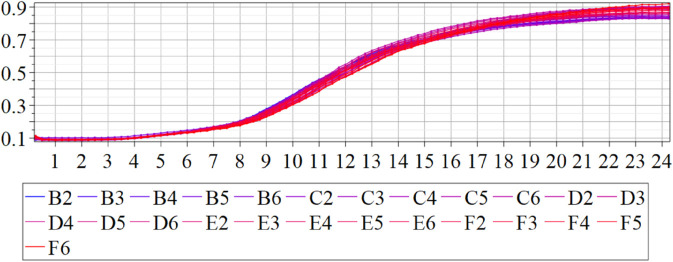
Original data, 33 ^∘^C.

**Fig 47:**
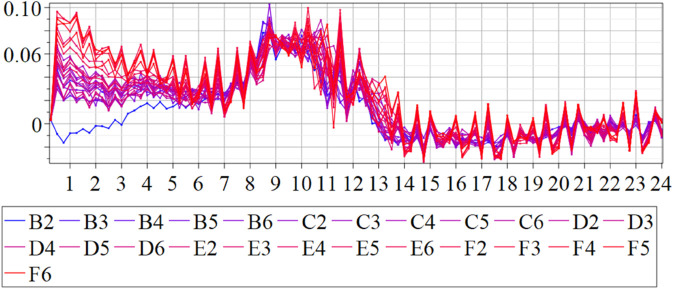
Fractional derivative with $\hat{\eta }$ = 1.2435, 33 ^∘^C.

**Fig 48:**
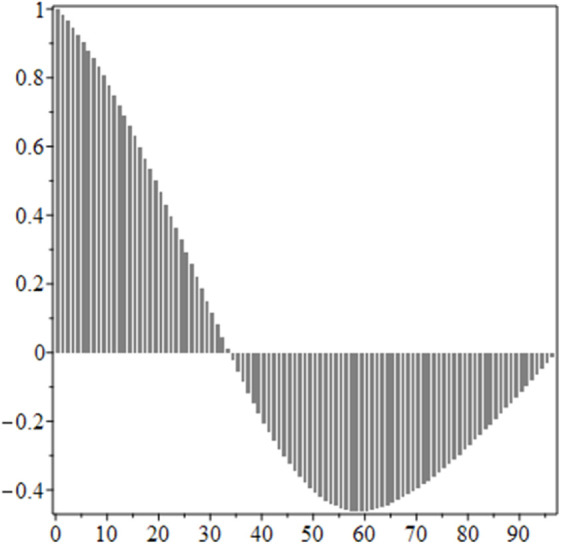
Autocorrelation of mean, 33 ^∘^C.

**Fig 49:**
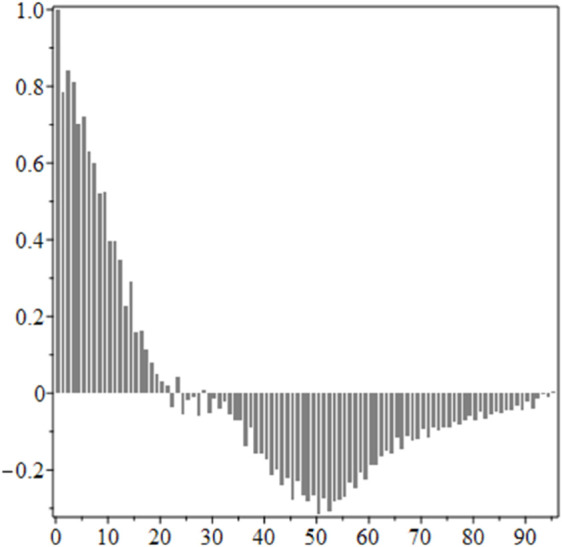
Autocorrelation of mean for fractional derivative with $\hat{\eta }$ = 1.2435, 33 ^∘^ C.

## Supporting information

**S1 Data File**

The minimal underlying data set for all findings described in the manuscript is provided in this file, which is in a compressed .rar format.
